# Instability of prevailing small molecule acceptors in organic solar cells toward water/nucleophiles

**DOI:** 10.1126/sciadv.aed7732

**Published:** 2026-04-22

**Authors:** Xiaowei Zhong, Xinzheng Yang, Jiyeon Oh, Xiaosong Li, Wei You

**Affiliations:** ^1^ Department of Chemistry, University of North Carolina, Chapel Hill, NC 27599, USA.; ^2^ Department of Chemistry, University of Washington, Seattle, WA 98195, USA.

## Abstract

Small molecule acceptors (SMAs) with A-D-A structures have become the key constituents in organic solar cells (OSCs); however, many of them have demonstrated instability toward external factors (including light, heat, water, and oxygen). This work explores the chemical reactivity of the double bond linking the donor (D) and acceptor (A) units in these molecules. Using a model compound, T4CN, constructed by a thiophene (T) and a strong acceptor moiety {2,2′-[1*H*-indene-1,3(2*H*)-diylidene]dimalononitrile, 4CN}, we show that water can break the double bond. The rate of this reaction depends on the electronic properties and steric hindrance. We found that common SMAs, including ITIC and Y6, react with nucleophiles such as amines and hydroxide, creating unintended products. These reactions can affect device performance and long-term stability. Among the tested materials, Y6, with its β-position side chains, showed the best resistance to water-induced degradation. Our findings highlight key factors affecting SMA stability and offer insights into designing more robust materials for future development of OSCs.

## Teaser

Strong push-pull effects make small molecule acceptors of organic solar cells chemically unstable.

## INTRODUCTION

With continued innovations in materials and devices, the efficiency of bulk heterojunction (BHJ)–based organic solar cells (OSCs) has continued to rise with record high numbers now breaching 20% (*1*–*4*). In the meantime, substantial efforts toward understanding and improving the intrinsic (in)stability of BHJ solar cells under environmental stressors (such as heat and light) have also generated notable progress (*5*–*10*). For example, Ade and co-workers applied the Flory-Huggins theory to construct a framework to address the morphological (in)stability of the BHJ, as BHJ kinetically quenched an electron-donating organic material and an electron-accepting one in a thermodynamically unstable thin film (*11*, *12*). On the other hand, Baran’s team recently elucidated the photodegradation pathways of polymer donors in BHJ OSCs with extensive outdoor testing (*13*).

**Fig. 1.**
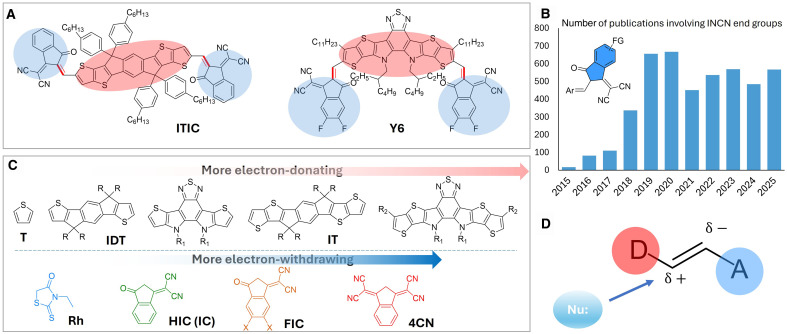
Polarized donor-acceptor double bonds in prevailing SMAs create sites susceptible to nucleophilic attack. (**A**) Structures of two representative SMAs, ITIC and Y6, respectively, both with D and A moieties connected by double bonds. (**B**) Increasing number of publications involving INCN unit as the terminal acceptor groups over the years. (**C**) Relative strength of electron-donating moieties and electron-withdrawing moieties. (**D**) Illustration of polarized double bonds; the δ+ carbon would be susceptible to nucleophilic attack.

A state-of-the-art BHJ OSC typically uses a donor polymer and a non-fullerene acceptor (NFA); the latter includes n-type polymers and small molecule acceptors [SMAs; typically fused ring electron acceptors (FREAs); (*14*–*17*)]. While the photoinduced instability of donor polymers (often chemical degradation) has been studied since the early days of BHJ OSCs (*18*–*20*), the emergence of NFAs has drawn research focus to the stability of NFAs, particularly SMAs. SMAs commonly adopt an A-D-A structure, where D is an electron-donating moiety (usually fused rings) and A (an electron-accepting moiety) is most commonly 2-(3-oxo-2,3-dihydro-1*H*-inden-1-ylidene)malononitrile (INCN) and its derivatives (*21*). Two representative SMAs, ITIC and Y6, are shown in Fig. 1A. The popularity of INCN and its derivatives is evident, with increasing number of publications that involve these building blocks every year (Fig. 1B). Generally, A and D are connected via a double bond typically formed through an aldol condensation. For most A-D-A–type SMAs, the double bond between A and D is characterized by a chemical shift around 8.5 parts per million (ppm), indicative of high deshielding of electron density (versus the usually observed chemical shifts at 4.7 ppm for most alkene protons). While most double bonds are quite stable, research on the stability of SMAs has shown that the double bond in such D-A structures is prone to metathesis (*22*, *23*), photoinduced isomerization (*24*) and cyclization (*25*), as well as degradation (*26*).

In 2018, Zhou and co-workers demonstrated the degradation of ITIC when it was in contact with polyethyleneimine (PEI), a common material used as electron transport layer (ETL) in OSCs (*27*); as a result, devices using ITIC with PEI as ETL yielded poor results. Subsequent investigations by the same team examined the effects of acidity and basicity on ITIC (*28*). While acetic acid had a negligible effect on ITIC, sodium hydroxide (NaOH; a strong base) caused extensive destruction of the molecule. These studies highlighted the potential reactivity of SMAs with interfacial materials (fig. S1). Another common ETL, zinc oxide (ZnO), can also degrade SMAs [including ITIC (*29*) and IT4F (*26*)]; the formation of aldehydes (retro-aldol condensation products) was observed by high-resolution mass spectrometry analysis. Further investigations by the same group examined more SMAs (ITIC, Y6, and N3) on ZnO, and the aldehyde formation was observed for all studied SMAs after 10 hours of irradiation (*30*). The researchers proposed that ZnO, under irradiation, can produce hydroxyl radicals that react with SMAs, leading to retro-aldol condensation. In addition, they observed that SMAs with β side chains were less affected by this degradation process.

In addition to the ETL-triggered decomposition of SMAs, Marks and co-workers reported the end-group interchange in a series of ITIC-based SMAs (same electron-donating moiety, with different end groups) in solution (fig. S2) (*31*). Specifically, when PM6, ITIC-0F, and ITIC-4F were mixed in a chloroform solution, a new species—ITIC-3F—was found. Notably, this end-group scrambling reaction was observed even with pure compounds in solution. For example, when pure ITIC-2F was dissolved in chloroform, equal amounts of ITIC-4F and ITIC-0F were formed (2 ITIC-2F ⇌ ITIC-4F + ITIC-0F). Because no aldehyde was detected in the scrambling/end-group exchange reaction, they proposed a [2 + 2] cycloaddition/cycloreversion mechanism based on the isotope-labeled nuclear magnetic resonance (NMR) study. More recently, Forrest and co-workers further demonstrated that two different SMAs (Y16 and ITIC) could also undergo end-group scrambling to produce a mixture of up to six different SMAs (*32*). This scrambling process appeared to be facilitated by preexisting trace amount of water and heat; the presence of the mixed SMAs had a strong impact on the device performance/reproducibility and long-term stability of the ternary blend-based OSCs.

**Fig. 2.**
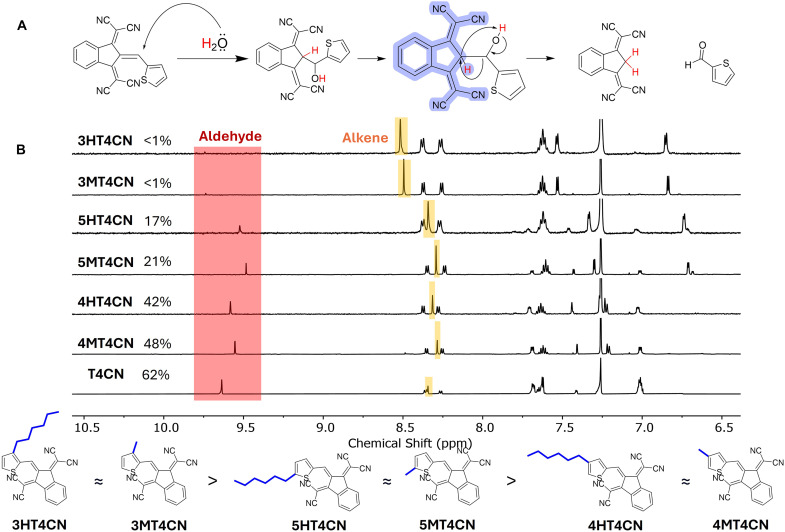
Steric hindrance near the D-A double bond suppresses water-induced cleavage in T4CN. (**A**) Proposed reaction mechanism. (**B**) NMR spectra were taken 3 hours after the solvent was added; no stirring during the reaction. Concentration: 2.5 mM (CDCl_3_/MeOD/D_2_O: 6/5/1, v/v/v).

These prior studies underscore the importance of the (in)stability of prevailing SMAs (e.g., Y series), the key component in the BHJ OSCs of record-high efficiency numbers, and point to the plausible root cause: the double bond between D and A moieties. However, double bond is a very common linker in conjugated polymers [e.g., poly(phenylene vinylene), PPV] and related small molecules (e.g., stilbene), yet PPV and stilbene are very stable toward nucleophiles (such as water). We hypothesize that the D-A structure (in SMAs) would induce the polarization of the double bond and thereby subject it to further reactions, and the susceptibility of the double bond to the degradation/reaction would be fundamentally determined by the relative strength of donor and acceptor moieties (Fig. 1C). Specifically, the δ+ carbon (next to the D moiety) is electrophilic and prone to nucleophilic attack by a nucleophile, whereas the δ− carbon (next to the A moiety) is nucleophilic. With a model D-A compound, T4CN (Fig. 2), we showed that water is the key factor that causes the decomposition of T4CN into corresponding D-aldehyde and the A moiety, and the rate of decomposition is highly affected by both electronic and steric effects in the D-A molecule. Further examinations of prevailing D-A–based SMAs revealed that these D-A molecules are sensitive to other nucleophiles as well (i.e., amine, hydroxide, and other INCN end groups) and even some electrophiles (e.g., aldehyde), indicating the general instability of the double bond in D-A–based SMAs. We further tested selected prevailing SMAs in the solid state at simulated 85°C/85% relative humidity (85/85) condition and found that Y6, with the β side chain, exhibited the best chemical stability to water.

## RESULTS

### Model study: Steric effect

According to our hypothesis, the stronger D (and A), the more polarized the double bond should be. Thus, we chose a common donor moiety (thiophene, T) and a strong acceptor moiety {2,2′-[1*H*-indene-1,3(2*H*)-diylidene]dimalononitrile, 4CN} to construct the model compound, T4CN, as the starting point. Not unexpectedly, this molecule decomposes readily in alcohol (with water) (fig. S3). To understand the mechanism, we conducted additional experiments with deuterated methanol (MeOD) and/or deuterated water (D_2_O) (fig. S4). Deuterated 4CN was obtained when using D_2_O, suggesting that water was the key participant in the decomposition reaction via the proposed retro-aldol condensation that generated the thiophene-aldehyde and the 4CN unit (Fig. 2A). Since the same decomposition reaction could also happen in chloroform (open air) albeit slowly under vigorous stirring, we proposed that the observed faster decomposition in alcohol can be ascribed to the dual role of alcohol: (i) dissolving water and (ii) facilitating proton transfer.

We next anchored alkyl chains to the different positions on thiophene of T4CN to explore the impact of steric hindrance on the decomposition (Fig. 2B and figs. S5 to S11). In situ NMR was used to monitor the decomposition of these molecules without stirring. As the reference, 62% of T4CN decomposed after 3 hours; by contrast, 3HT4CN and 3MT4CN (where sides chains are closest to the double bond, at the 3 position of the thiophene, thereby offering the strongest “protection” effect) exhibited essentially no decomposition after 3 hours and only showed about 20% decomposition after 24 hours. Shifting the side chains to the 4 or 5 position of the thiophene would diminish the steric “protection”; thus, the corresponding molecules (e.g., 4HT4CN and 5HT4CN) exhibited appreciable decomposition after 3 hours. However, it is worth noting that anchoring side chains on the 5 position offers higher stability than on the 4 position (i.e., the former molecules having less decomposition). Since the side chains in 4 and 5 positions are similarly farther away from the double bond, the less decomposition observed for 5MT4CN (5HT4CN) implies a weak yet effective delocalization between the ortho-alkyl substituent (5 position) and the π system, thereby decreasing the reactivity of the double bond toward water. These results clearly show that steric hindrance can have a notable impact on the stability of the double bond, with the alkyl chain (offering the steric hindrance; on the D moiety) next to the double bond offering the best protection against decomposition induced by the nucleophilic attack. It is also interesting to observe that even one methyl group is already sufficient (as good as the bulkier hexyl) to enable the steric “protection” effect. Nevertheless, the intrinsic stability of the double bond of D-A–based T4CN would eventually lead to the decomposition, even with the proper steric protection (i.e., in the case of 3MT4CN and 3HT4CN).

### Extended set of SMAs: Electronic effect

**Fig. 3.**
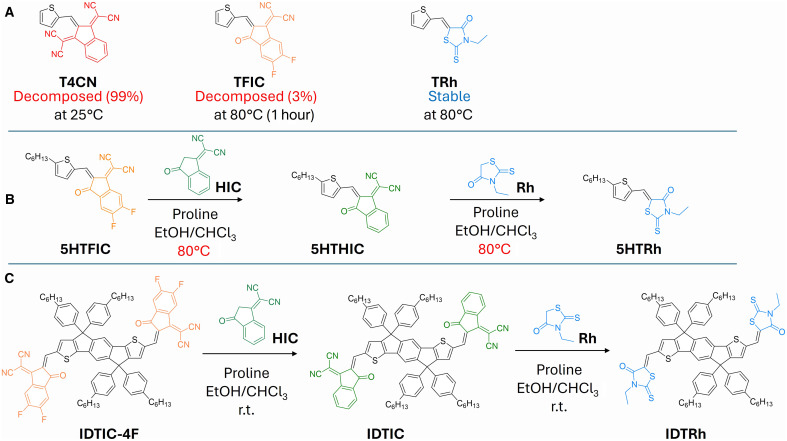
Donor and acceptor strength control the stability of D-A double bonds. (**A**) Model compounds in water-containing environments. The decomposition rate is affected by D-A strength. (**B**) Thiophene-based D-A molecules would undergo end-group exchange under condensation condition at elevated temperature. (**C**) IDT-based D-A molecules would undergo end-group exchange under condensation condition at room temperature (r.t.).

In theory, the electronic strength of D and A would decide the level of polarization of the double bond in between D and A moieties. As 4CN moiety is a very strong acceptor, we chose two weaker acceptor moieties, fluorinated IC (FIC) and rhodanine (Rh), to test the hypothesis (Fig. 3A). While T4CN decomposed completely in ethanol at room temperature within an hour under stirring, TFIC {(Z)-2-[5,6-difluoro-3-oxo-2-(thiophen-2-ylmethylene)-2,3-dihydro-1*H*-inden-1-ylidene]malononitrile} was stable at room temperature in ethanol (water-containing environment) but decomposed slowly at 85°C (fig. S12). By contrast, TRh [(Z)-3-ethyl-5-(thiophen-2-ylmethylene)-2-thioxothiazolidin-4-one; with the least electron-deficient moiety Rh in this series] was stable even at 85°C (fig. S13). We also changed the electron-donating units to pair with the HIC [2-(3-oxo-2,3-dihydro-1H-inden-1-ylidene)malononitrile; also known as INCN] unit, all of which showed decomposition at different extent under heating in ethanol (fig. S14).

To further explore the relative stability of the above series, we conducted the possible end-group exchange reactions. When mixing 5HTFIC with HIC (a weaker A moiety than the FIC), no exchange reaction was observed at room temperature under the condensation condition; however, increasing the temperature to 80°C promoted a complete end-group exchange (Fig. 3B and fig. S15). Similarly, 5HTHIC underwent the end-group exchange with Rh to form 5HTRh that should have a more stable double bond. Switching the D moiety from thiophene to indacenodithiophene (IDT), a more electron-donating unit, the very same end-group exchange reactions now occurred readily at room temperature (Fig. 3C and fig. S16). These results suggest that IDT, a stronger donor moiety, could cause a less stable double bond in its D-A–based SMAs than the thiophene-based ones. Notably, there is no aldehyde observed in in situ NMR reaction (fig. S17), which implies that such exchange reaction would not need to go through the aldehyde as the intermediate, as Marks and co-workers also observed (*31*). In addition, we find that IDTIC could also react with thiophene-2-carboxaldehyde (an electrophile) under our test conditions (fig. S18). Thus, the double bond (in between D and A) could exhibit both electrophilicity (i.e., the δ+ carbon, next to the D moiety) and nucleophilicity (i.e., the δ− carbon, next to the A moiety), with the reactivity controlled by the relative strength of the D and A moieties. These end-group exchange results allow us to qualitatively rank the relative strength of these D and A moieties (Fig. 1C). According to our hypothesis and experimental results, for example, linking a strong donor and strong acceptor via a double bond in an SMA would result in pronounced instability and easy decomposition, whereas pairing a weak donor and a weak acceptor would lead to increased stability.

If the double bond is susceptible to the nucleophilic decomposition by water, other nucleophiles would also be able to decompose the SMAs with similar mechanisms. When we tested the stability of selected SMAs with amine and hydroxide, two other commonly encountered nucleophiles in fabricating BHJ devices, we observed the expected decomposition of SMAs. For example, treating BTP-4F with NaOH (aqueous) led to instant disappearance of the color of the BTP-4F. NMR revealed the product to be the hydroxide adduct, manifested by the noticeable up-shift of the adjacent hydrogen on benzene (fig. S19). While this adduct would decompose a week later as indicated by the color change, adding water into the fresh solution of this adduct to reduce the basicity would recover BTP-4F, suggesting the reversible nature (equilibrium) of aldol condensation (fig. S20). Furthermore, neutralizing this adduct (when it was freshly prepared) with acid would also recover BTP-4F. In another experiment, adding excess ethanolamine to a solution of IDTIC solution instantly decomposed the IDTIC (i.e., bleaching of the solution), and NMR revealed the product to be an imine (fig. S21). Since imine is rather unstable, aldehyde was formed a day later from the imine decomposition. If ammonium salt (or perovskites, which contain ammonium salts) is added to the IDTIC solution, no reaction happens, indicating that the low pH can suppress these reactions (fig. S22).

### Mechanism

**Fig. 4.**
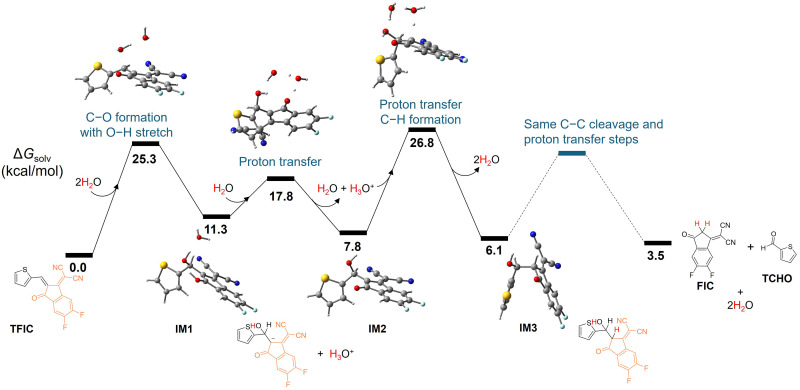
DFT simulations reveal a water-assisted nucleophilic addition pathway for D-A double-bond cleavage. DFT[PBE0/GD3(BJ), IEFPCM, MeOH] predicted energy surface for the proposed reaction pathway of TFIC via direct deprotonation.

We applied density functional theory (DFT) simulation to study the decomposition mechanism of four representative D-A molecules, T4CN, TFIC, TRh, and 3MT4CN, to understand the electronic and steric effects (figs. S23 to S26). Figure 4 presents the reaction pathway for TFIC, indicating that the H_2_O addition on the double bond is solvent-dependent. Specifically, a water molecule first nucleophilically attacks the double bond to form the C─O bond with O─H cleavage to form IM-1. This intermediate will undergo deprotonation and protonation to reach a lower-energy IM-3, which would potentially undergo C─C bond cleavage and further proton transfer to offer FIC and TCHO as the degradation products. Thus, the solvent will be crucial for the decomposition as it will affect the proton transfer, and excess water molecules in the system will facilitate the proton transfer (fig. S23A). Compared with the direct water addition to the double bond, this water-assisted pathway has a lower energy barrier (from 48.1 to 25.3 kcal/mol) (fig. S23B).

Because of the different steric and electronic impact from the four molecules in the simulation, the energy barrier to form the C─O bond (from the water molecule attacking the double bond) follows the trend of TRh > 3MT4CN > TFIC > T4CN. This trend well matched our observation, with TRh giving the highest stability (fig. S24), T4CN showing rapid (fig. S25, A and B) cleavage, and 3MT4CN (with methyl side chain) (fig. S26) and TFIC (with medium strength acceptor, FIC) slowing down the decomposition. Proton transfer was the key step for all three key steps: C─O bond formation, O─H bond cleavage, and C─H bond formation. As proton transfer is the most important external factor to account for the decomposition of the double bond, the solvent and the water concentration would thereby affect the decomposition rate. Notably, the intermediate of T-Rh-H_2_O adduct is not found due to its high energy, which could explain the high stability of TRh. On the other hand, for both 3MT4CN and T4CN, the energy values of products (aldehydes and 4CN) are lower than those of the reactants (−0.9 kcal/mol for 3MTCN and −1.6 kcal/mol for T4CN, respectively); thus, the degradation is thermodynamically favored. Although TFIC has a lower energy barrier for C─O bond formation compared to 3MT4CN (25.3 versus 26.6 kcal/mol), heating is required to decompose TFIC into aldehydes while 3MT4CN slowly decomposes completely at room temperature. This can be explained by the higher energy of the products in the case of TFIC than those of 3MTCN (3.5 versus −0.9 kcal/mol).

### Solid state testing (85/85)

While solution-based experiments are crucial to elucidate the mechanism for the decomposition of SMAs, it would be most practically relevant to conduct the stability testing in the solid state, ideally under 85/85 condition. To accelerate the testing, we drop-cast SMAs on glass substrate and stored samples without any encapsulation under a homemade chamber of simulated 85/85 condition (fig. S27). Every week, one sample was retrieved to dissolve the SMA, which was then characterized by NMR to estimate the percentage of decomposition (based on the ratio of integration between the aldehyde and the double bond).

We first tested the model compound, T4CN, which decomposed completely after just 1 day (fig. S28). When polystyrene was used to encapsulate the T4CN film, it still reached complete decomposition after 3 days. These results on T4CN highlight the fact that while encapsulation can certainly slow down decomposition (depending upon the water permeability), the intrinsic instability of double bond in T4CN would eventually lead to the decomposition.

**Fig. 5.**
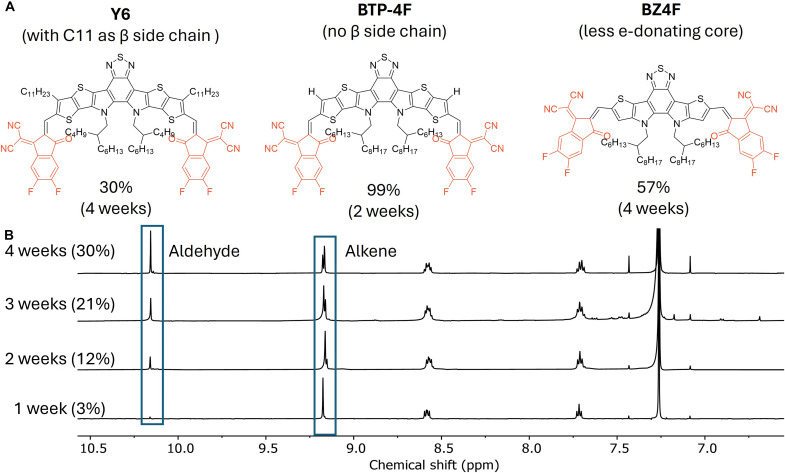
β side chains enhance SMA stability under 85°C/85% relative humidity solid-state conditions. (**A**) Three Y6-based SMAs tested under 85/85, with percentage of decomposition after given time. (**B**) The time-dependent decomposition study of Y6 under 85/85.

We then evaluated a series of Y6-based SMAs (Fig. 5). With β side chain sterically protecting the labile double bond, Y6 was quite robust under our testing condition, with 30% decomposition after 4 weeks. By contrast, BTP-4F, structurally identical to Y6 but lacking the β side chain, completely decomposed after 2 weeks (fig. S29). Removing one thiophene unit from each side of the donor core of BTP-4F leads to BZ4F, which has a less electron-donating core and thereby less polarized double bond; consequently, BZ4F can better withstand the decomposition (57% decomposed after 4 weeks) (fig. S30). These results indicate that the β side chains can more effectively protect the double bond (even though it is more polarized in Y6 than in BZ4F).

We also tested the IDT series. IDIC-4Cl showed 32% decomposition after 2 weeks (fig. S31); switching the end group to HIC (less electron-withdrawing) could slow down the decomposition, as aIDIC (an IDIC analog with asymmetrical side chains) showed a similar extent of decomposition (32%) only after 4 weeks (fig. S32). On the other hand, IT4F, with stronger electron-donating moiety and also stronger electron-accepting moiety, decomposed at much faster rate, which is 34% decomposition after just 1 week (fig. S33).

The test results of these different SMAs in the solid state under our simulated 85/85 corroborate the decomposition behavior of SMAs in the solution (Figs. 2 and 3). However, the instability of the double bond between D and A in these SMAs presents an interesting dilemma. Most SMAs are designed to absorb longer wavelengths and thereby use rather strong donor moieties and strong acceptor moieties to achieve a small bandgap, yet this design increases the risk of decomposition/degradation of the SMAs via the more polarized double bond. The ultimate solution is perhaps the removal of the double bond between D and A, and connecting D and A via single bonds (*33*–*35*) or ring fusion (*36*, *37*); however, none of the approaches have delivered SMAs that can rival the double bond–containing ones (e.g., Y6) in achieving high efficiency (over 17%). As a mitigating approach, we propose a “gradient” design strategy where a relatively weaker donor (e.g., thiophene) is inserted in between a strong donor (e.g., BTP) and a strong acceptor (e.g., 4F). The newly created SMA, BTP-T-4F (structure in fig. S34), has a similar structure to that of BTP-4F (and Y6); however, BTP-T-4F exhibited a much higher stability than BTP-4F, showing 36% decomposition after 4 weeks under 85/85 (fig. S34), similar to Y6 tested under the same condition.

The active layer of a BHJ device is typically a thin film of ~100 nm that contains a blend of a donor polymer and an SMA, much thinner than the drop-cast film used in the previous stability test of pure SMAs. Thus, to examine how intrinsic chemical stability of SMAs correlates with the stability of the corresponding BHJ device, we selected five representative SMAs (fig. S42) to pair with a donor polymer (PM6 or D18-Cl) for a simplified device stability evaluation under 85/85 (fig. S43). Under this accelerated aging condition, all devices exhibited rapid performance decay, with most losing the majority of their power conversion efficiency (PCE) within 1 week. However, a distinct stability trend was observed among these five SMAs. Y6-based devices retained ~50% of their initial PCE after 1 week, whereas BTP-4F–based devices degraded most rapidly. BZ4F-based devices showed improved stability relative to BTP-4F–based ones, and incorporation of additional thiophene units (BTP-T-4F and BZ-T-4F) further enhanced the stability of their corresponding BHJ devices. This trend is consistent with our molecular-level hypothesis that modulation of donor strength and conjugation gradient could influence intrinsic chemical stability of SMAs. To directly probe the chemical origin of the degradation, each active layer of the aged devices was dissolved in chloroform and further analyzed via spectroscopic techniques. Ultraviolet-visible (UV-vis) absorption spectra (fig. S45) and fluorescence images under 365-nm excitation (fig. S46) revealed a progressive formation of the corresponding aldehyde species, consistent with a retro-condensation of the double bond between D and A. Notably, even Y6—designed for improved stability—indicated the aldehyde formation in Y6-based BHJ devices under the prolonged 85/85 exposure.

## DISCUSSION

We have experimentally verified that the prevailing SMAs are intrinsically unstable toward water/nucleophiles. The main reason resides in the highly polarized double bond in between the electron-donating and electron-accepting moieties in these SMAs. Adding steric hindrance next to the double bond could shield it from the nucleophilic attack and increases the stability of the SMAs (e.g., Y6), so does the use of weaker donor and acceptors in the design of SMAs (yet doing so would enlarge the bandgap, an undesirable consequence). While replacing the double bond with single bond or ring fusing could be the ultimate solution, one mitigating approach at this stage is to add a weaker donor in the design of SMAs to create a “gradient”; this approach could maintain the desirable low bandgap and energy levels for high efficiency OSCs, with much improved chemical stability of the double bond toward nucleophiles.

From the practical perspective, since the polarized double bonds in prevailing SMAs are highly reactive, extra care needs to be taken in choosing the interlayer [hole transporting layer (HTL)/electron transporting layer (ETL)] materials and processing conditions when using these SMAs for OSCs.

## MATERIALS AND METHODS

### General information

NMR measurements were recorded with the Bruker Avance III Nanobay 400-MHz NMR spectrometer and the Bruker Avance 500-MHz NMR spectrometer. For the thin film for the 85/85 study, 10 mg of SMAs was dissolved in 5 ml of chloroform. One milliliter of solution was drop-casted on 1-cm^2^ glass substrate.

### General synthesis

The series of T4CN molecules were synthesized through refluxing aldehydes and 4CN (1 to 1 ratio) in acetic anhydride (5 M) for 1 hour. After heating, the acetic anhydride was removed, and the product was purified with column chromatography using chloroform as the eluent. Other D-A molecules were synthesized using proline as the catalyst (*38*).

### Computational methods

All DFT (*39*, *40*) calculations were performed using Gaussian 16 quantum chemistry program package (*41*). The geometrical structures of the ground states of all complexes were optimized using the PBE0 functional (*42*, *43*) with the Def2-TZVP all-electron basis set for all atoms (*44*, *45*). The D3 version of Grimme’s dispersion with Becke-Johnson damping (*46*) was added to the PBE0 functional to account for the noncovalent interactions. The solvent effect was corrected using the integral equation formalism polarizable continuum (IEFPCM) solvation model (*47*) with the Solvation Model based on Density (SMD) atomic radii (*48*) for methanol (ε = 32.613). The accuracy of numerical integrations is at the ultrafine grid (99,590) level. Frequency and intrinsic reaction coordinate calculations at optimized structures confirm no imaginary frequency at all equilibrium structures and only one imaginary frequency connecting correct stationary points at each transition state. Thermal corrections were obtained at 298.15 K and 1 atm (1 atm = 101.325 kPa) pressure on optimized structures using harmonic potential approximations.

### Device stability test

All devices had a configuration of indium tin oxide (ITO)/ZnO/donor:SMA/MoO_3_/Ag. A thin ZnO electron-transport layer was prepared by spin-coating a precursor solution of zinc acetate in 2-methoxyethanol with ethanolamine onto cleaned ITO substrates at 3500 rpm for 60 s, followed by annealing at 200°C for 30 min. The substrates were then transferred into a nitrogen-filled glovebox. The active layer was spin-coated from a chloroform solution (17 mg ml^−1^, donor:acceptor = 1:1.2 by weight) at 2500 rpm for 60 s, followed by thermal annealing at 100°C for 10 min. The resulting films were stored under 85°C/85% relative humidity conditions for accelerated aging. At designated time points, the films were removed from the environmental chamber, and MoO_3_ (10 nm) and Ag (100 nm) electrodes were sequentially deposited by thermal evaporation under high vacuum. The completed devices had an active area of 6.8 mm^2^. After photovoltaic characterization, the active layer of each device was dissolved in 10 ml of chloroform, and the resulting solutions were used for UV-vis absorption and fluorescence analysis.
