# Supplementary material for: Instability of prevailing small molecule acceptors in organic solar cells toward water/nucleophiles
Source: Sci Adv. 2026 Apr 22;12(17):eaed7732. doi: 10.1126/sciadv.aed7732 (PMC13101862; doi:10.1126/sciadv.aed7732)
Supplement: Supplementary file 1 — Figs. S1 to S46 Table S1 [file sciadv.aed7732_sm.pdf]

Science Advances
AAAS

Supplementary Materials for

Instability of prevailing small molecule acceptors in organic solar cells toward water/nucleophiles

Xiaowei Zhong *et al.*

Corresponding author: Wei You, wyou@unc.edu

Sci. Adv. **12**, eaed7732 (2026)
DOI: 10.1126/sciadv.aed7732

This PDF file includes:

Figs. S1 to S46
Table S1

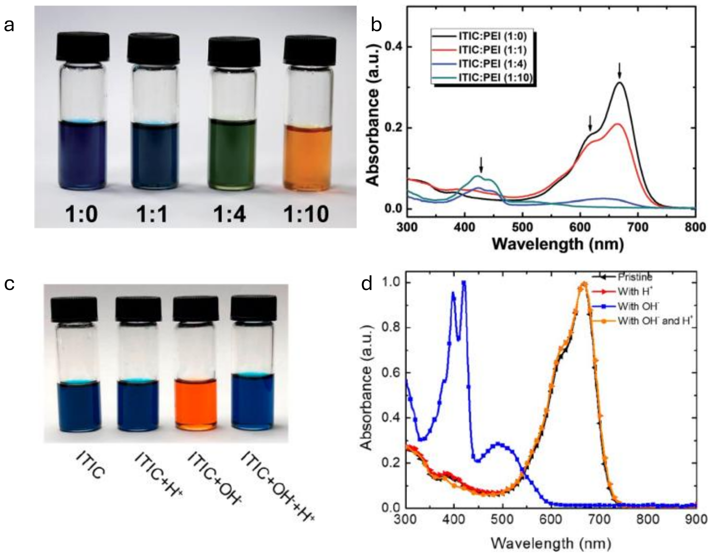

Figure S1. ITIC reacts with bases. (a) color change and (b) absorption change of ITIC when mixing with different equivalent of PEI. (c) color change and (b) absorption change of ITIC when adding HOAc or NaOH.

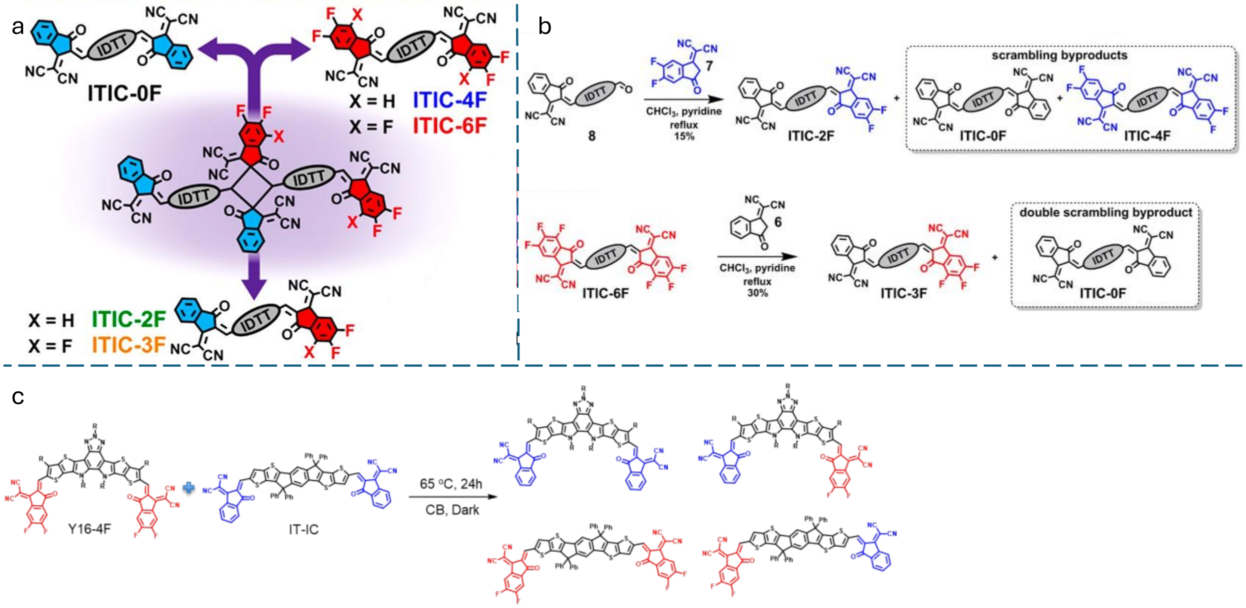

Figure S2. End group redistribution observed in ITIC derivatives. (a) proposed mechanism. (b) end group redistribution and exchange observed in synthesis. (c) End group redistribution could also happen with two SMAs with different electron-donating moieties.

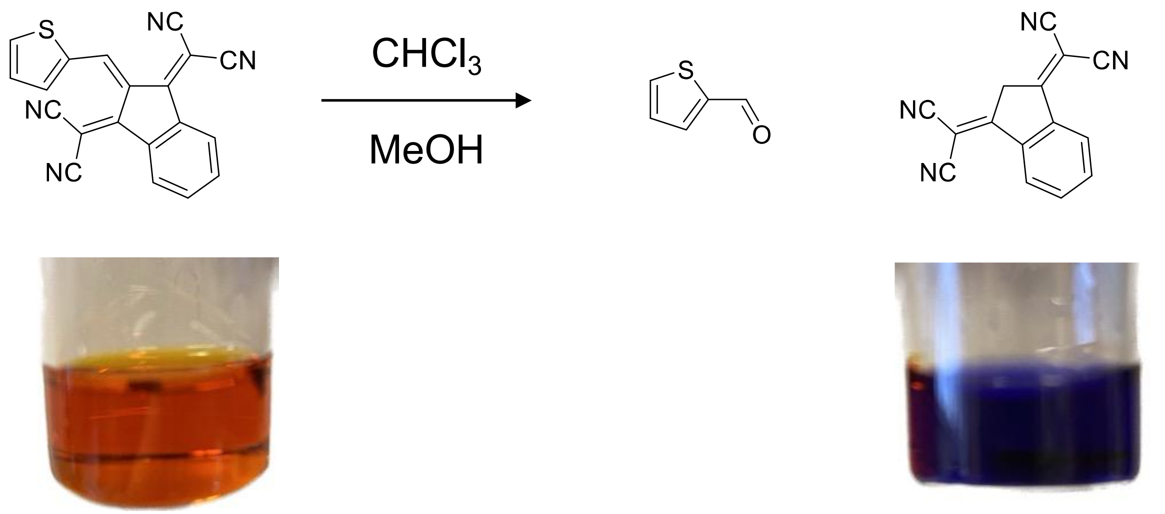

Figure S3. Color change of the cleavage of T4CN

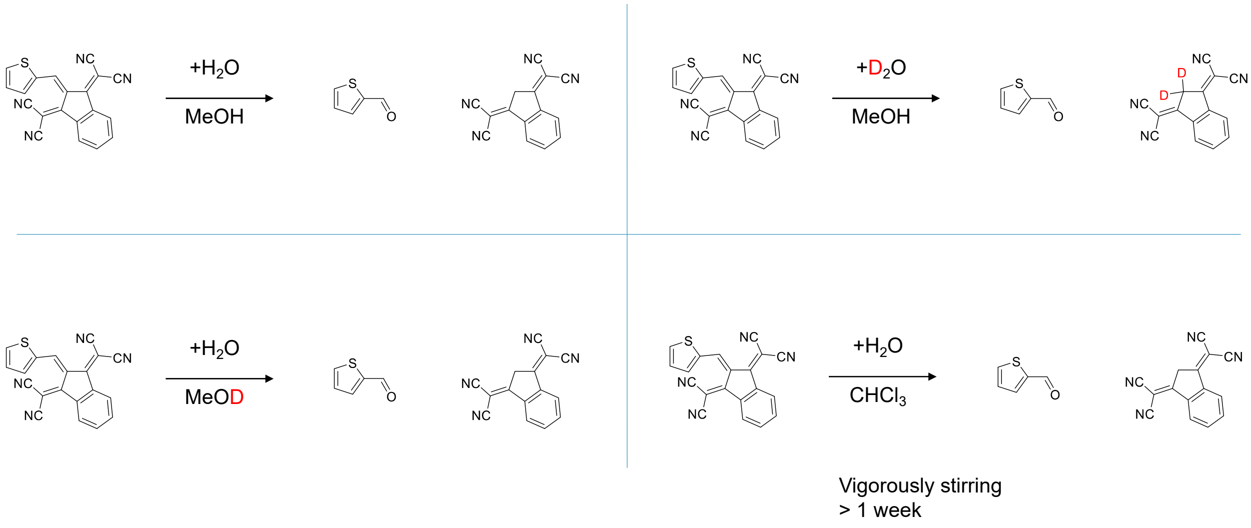

Figure S4. Deuterated experiments of T4CN

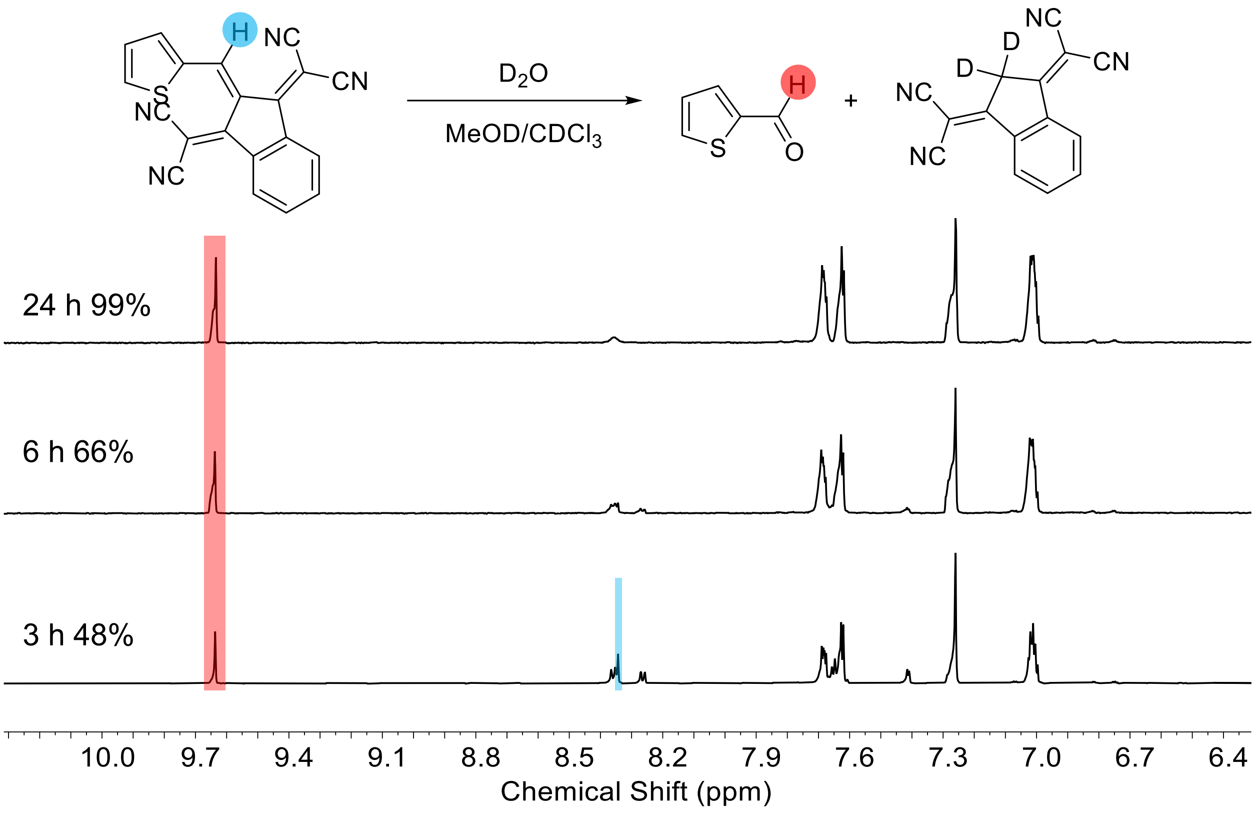

Figure S5. **NMR spectra of T4CN decomposition**

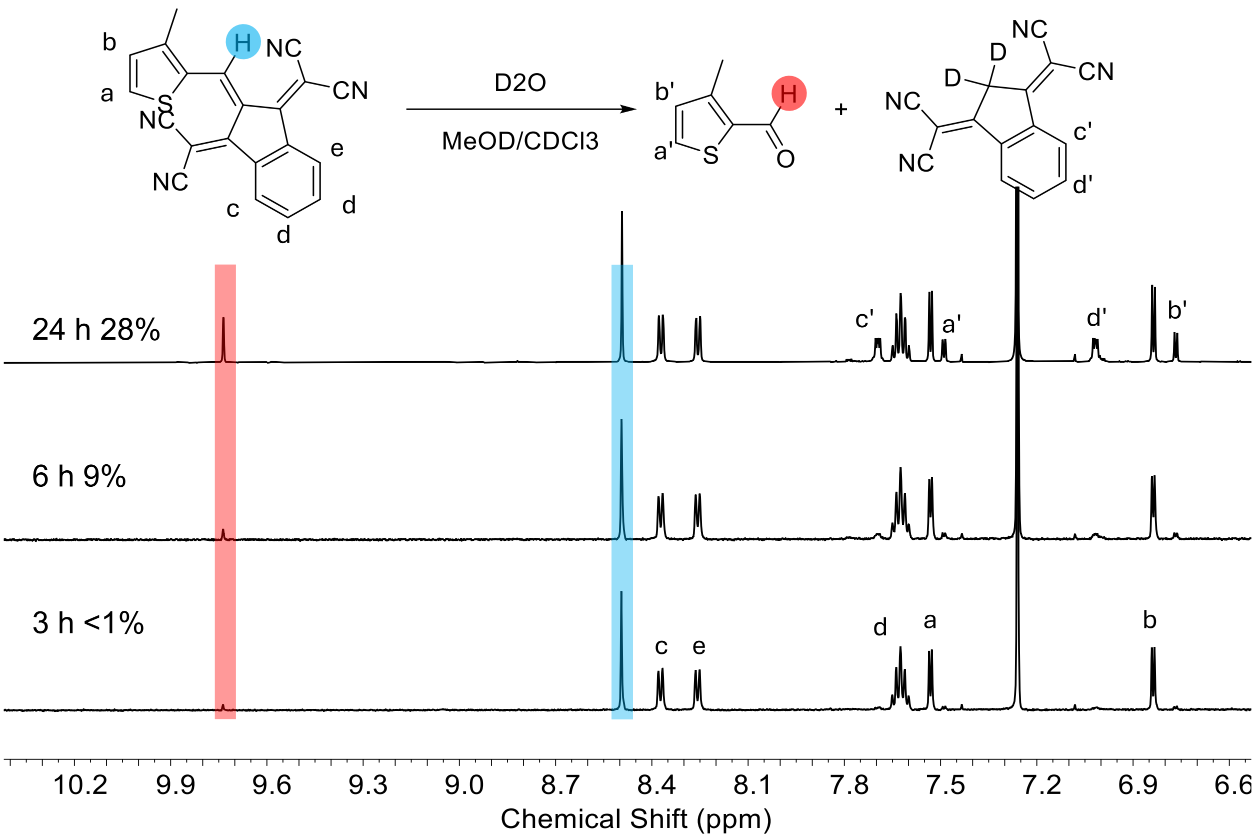

Figure S6. NMR spectra of 3MT4CN decomposition

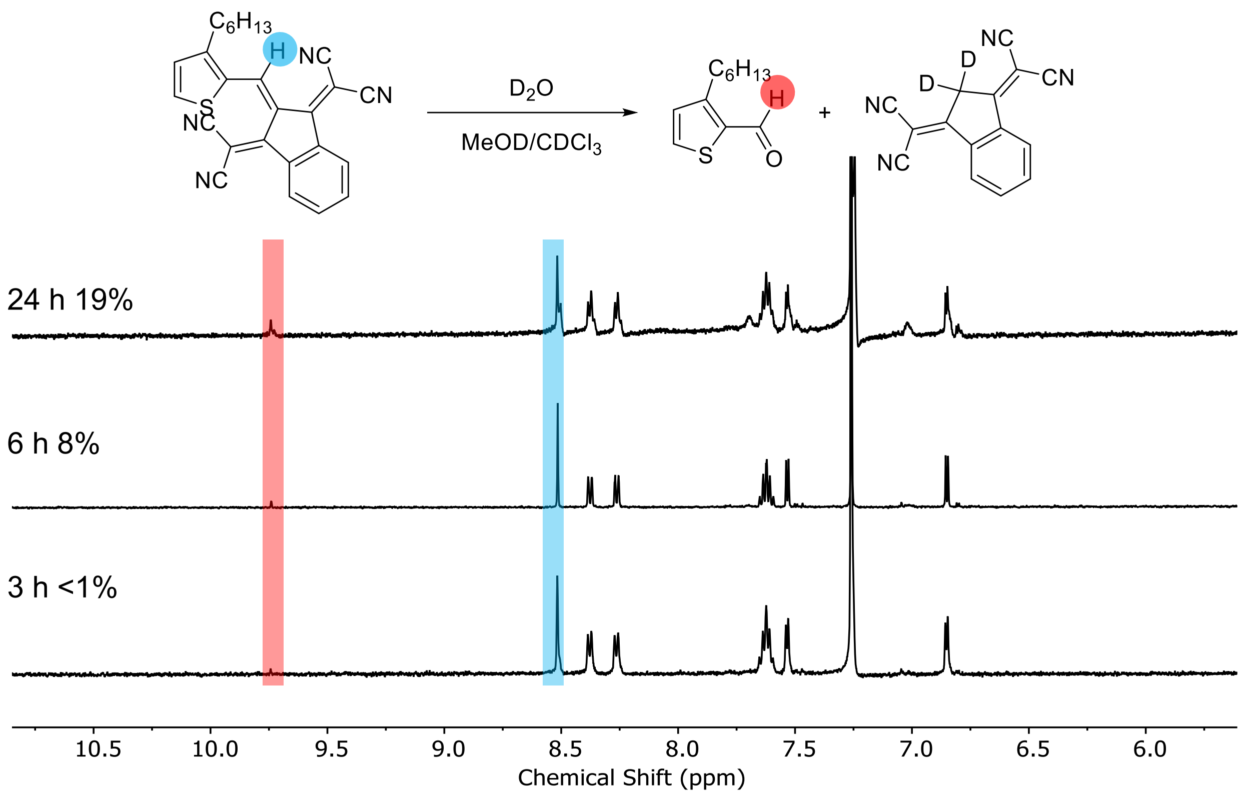

Figure S7. NMR spectra of 3HT4CN decomposition

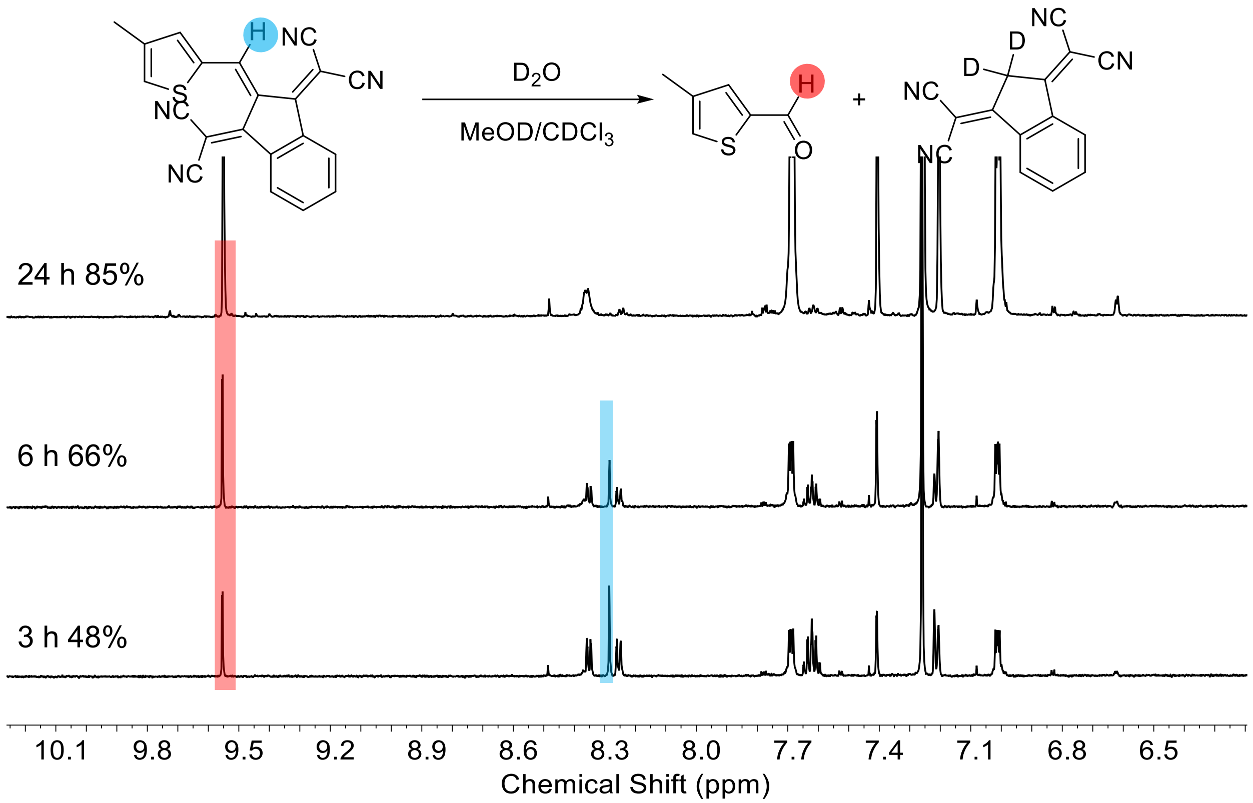

Figure S8. NMR spectra of 4MT4CN decomposition

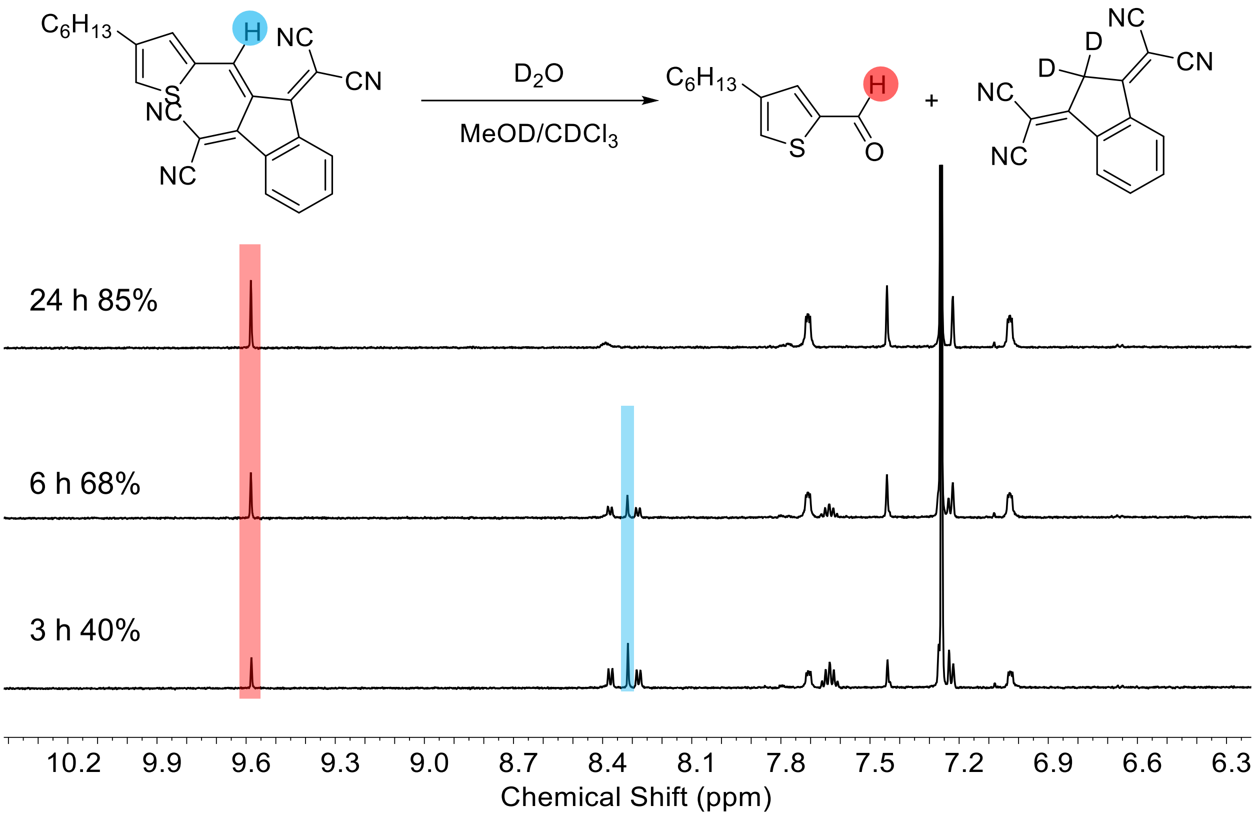

Figure S9. NMR spectra of 4HT4CN decomposition

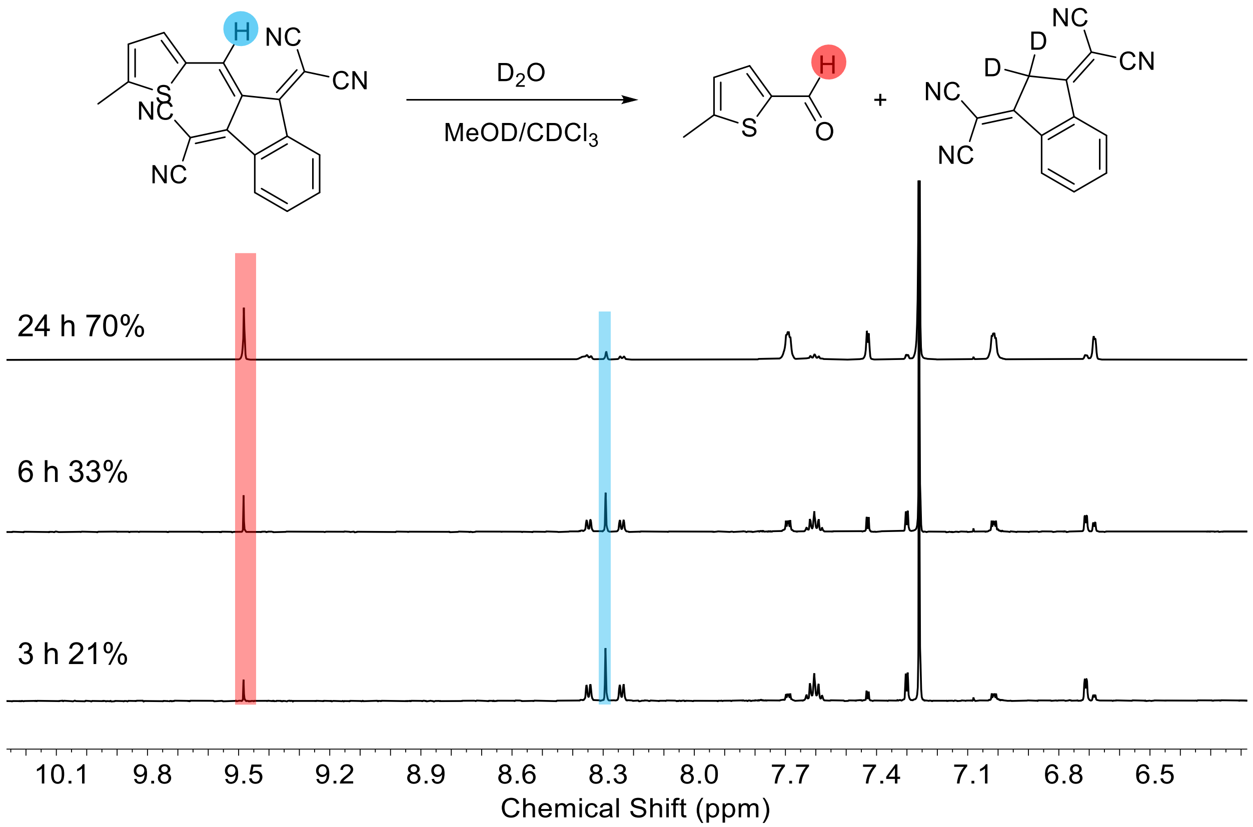

Figure S10. NMR spectra of 5MT4CN decomposition

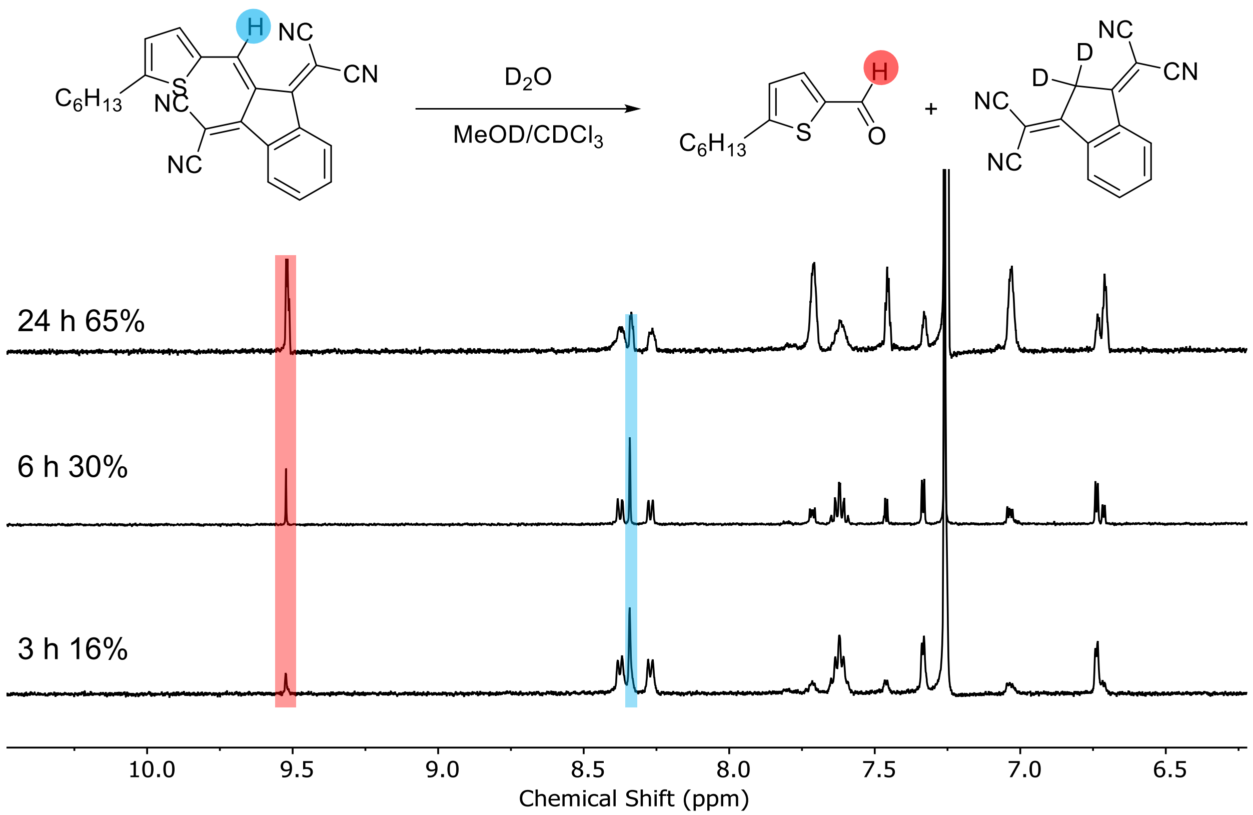

Figure S11. NMR spectra of 5HT4CN decomposition

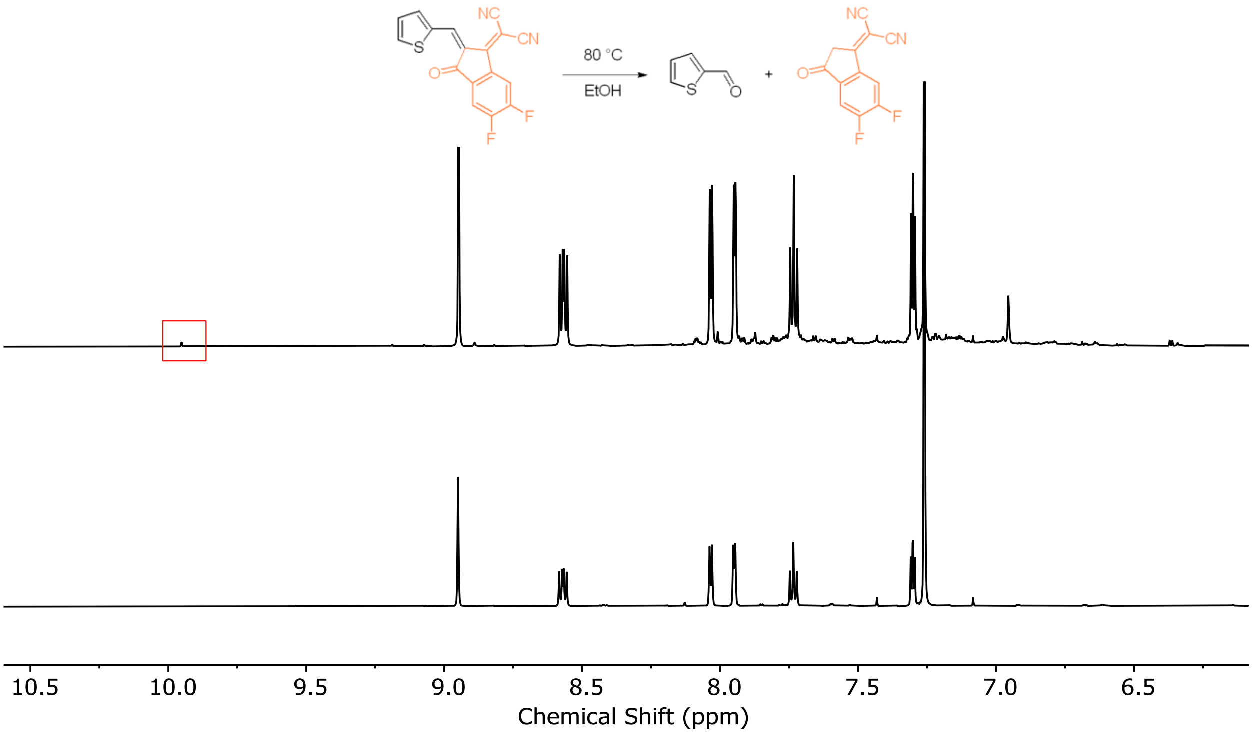

Figure S12. NMR spectra of TFIC before and after heating (1h), aldehyde peak is observed after heating.

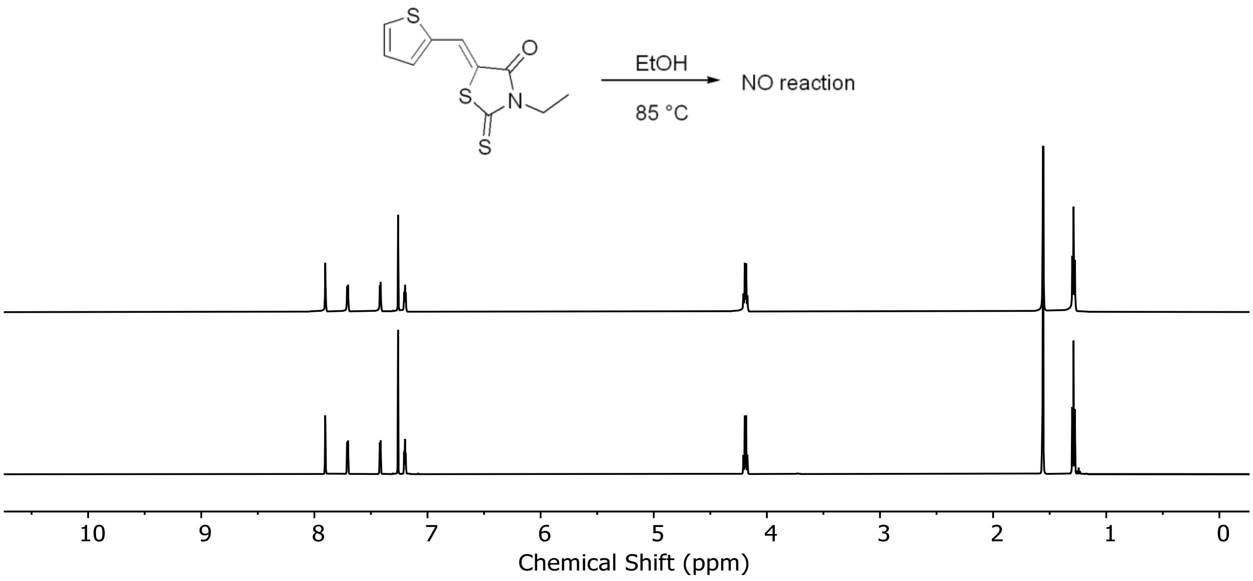

Figure S13. NMR spectra of TRh before and after heating

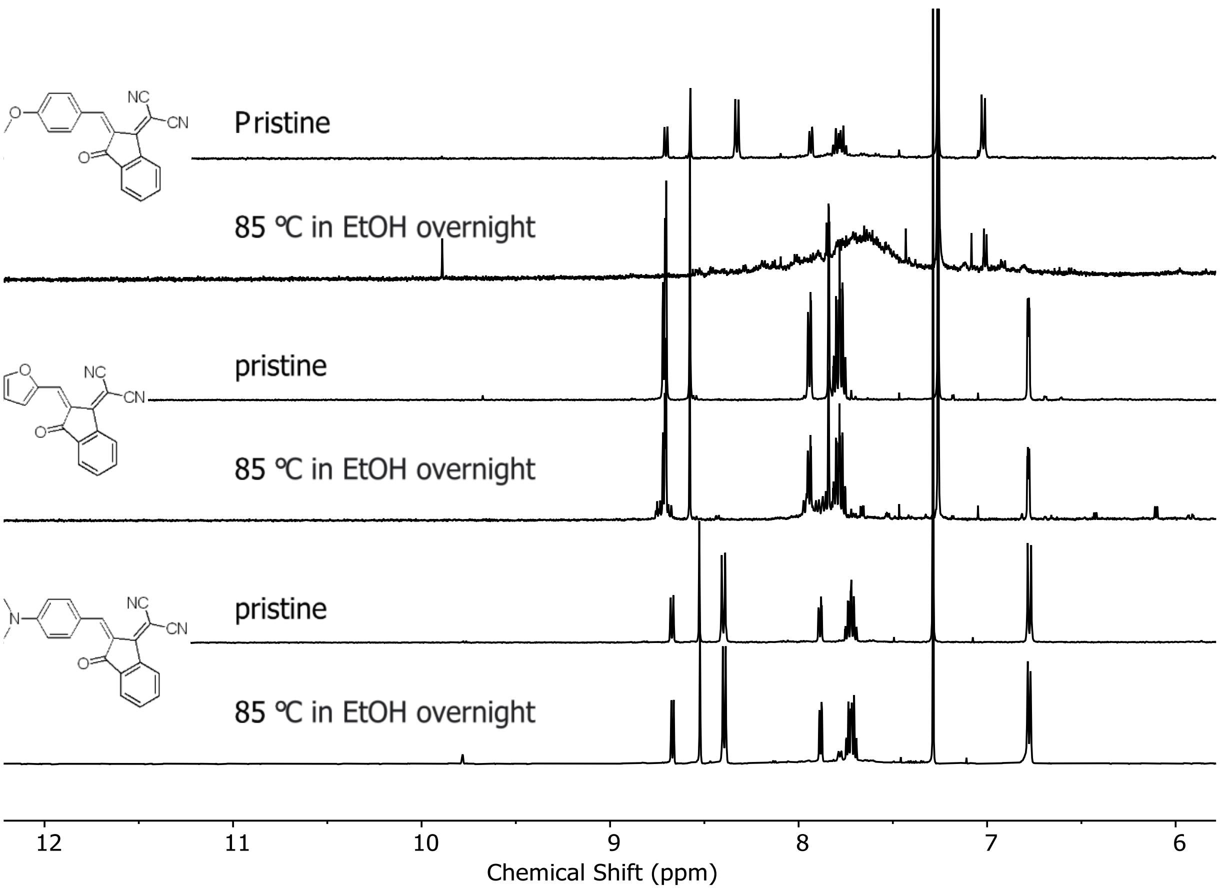

Figure S14. Stacked NMR spectra of three D-A molecules before and after heating.

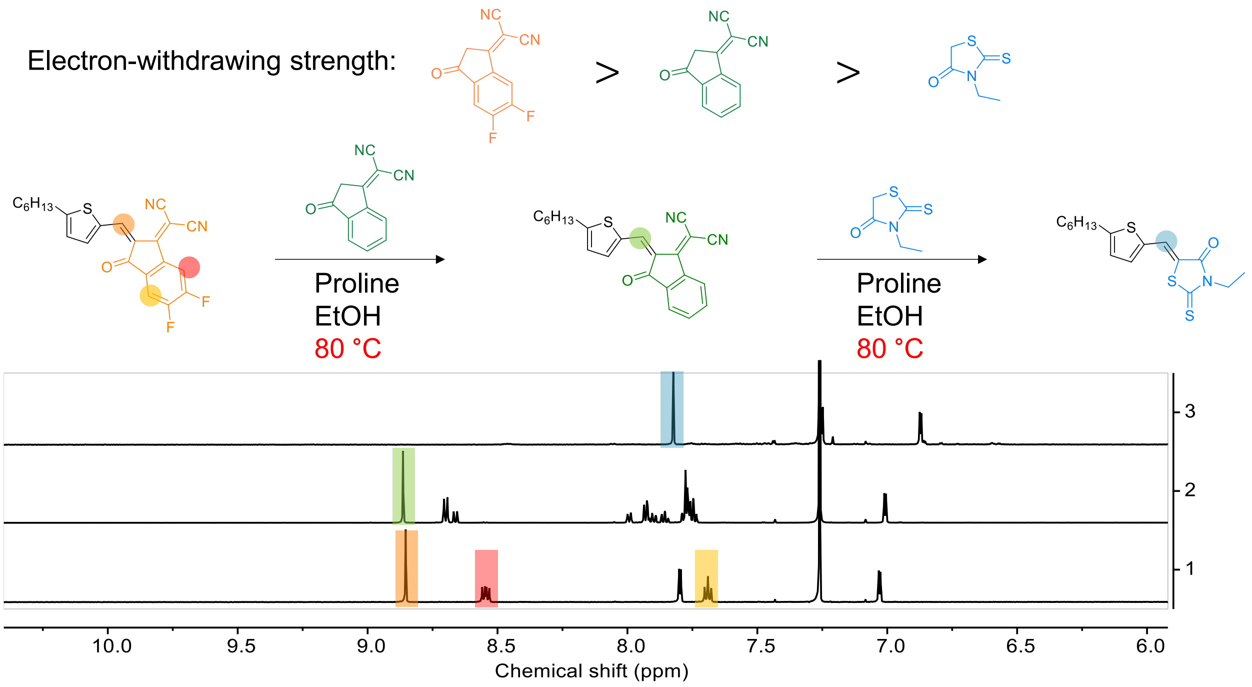

Figure S15. End group exchange with thiophene as the electron-donating unit.

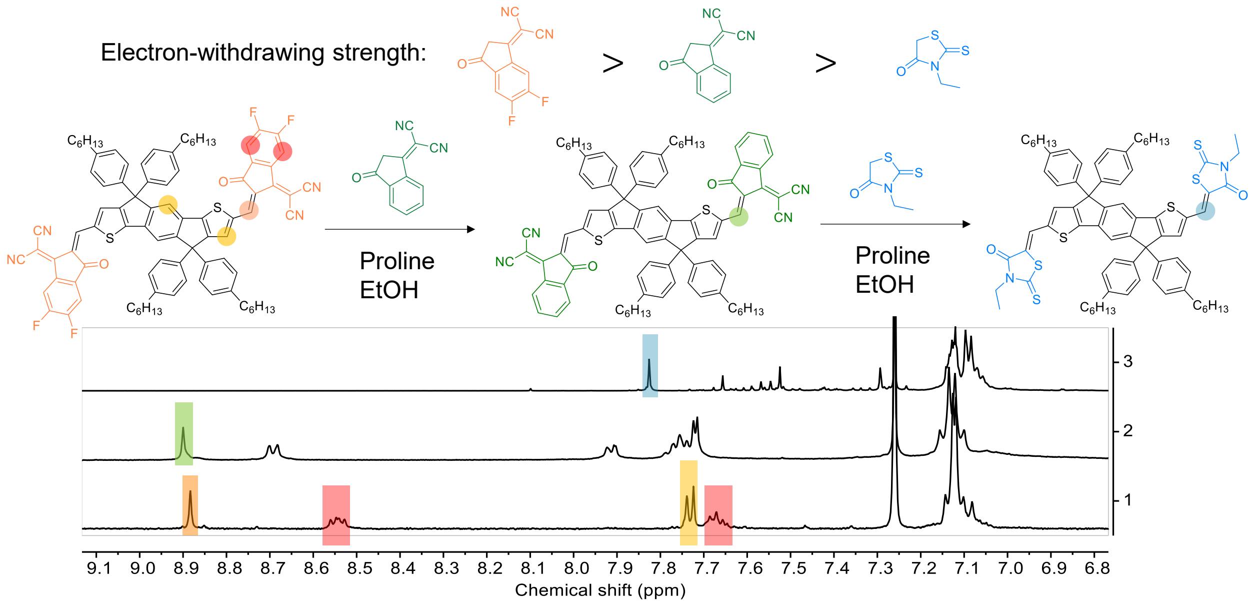

Figure S16. End group exchange with IDT as the electron-donating unit.

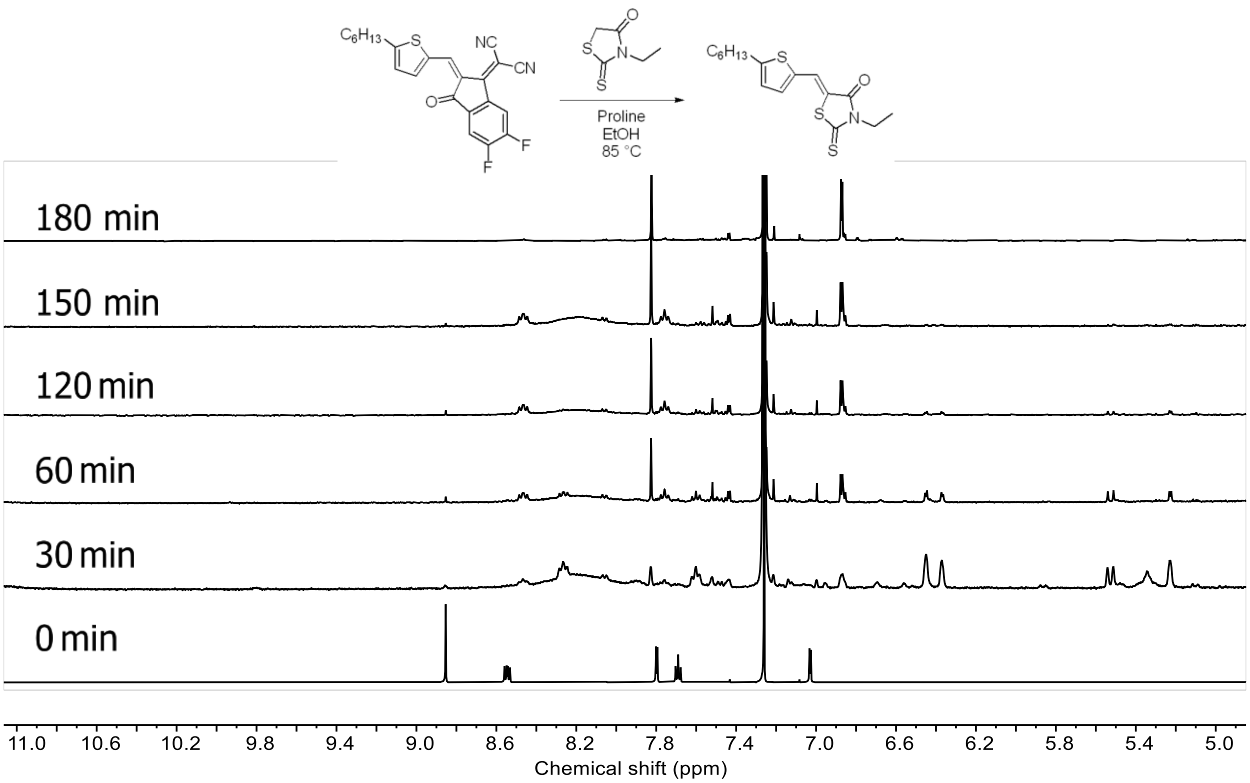

Figure S17. In-situ NMR of the end group exchange.

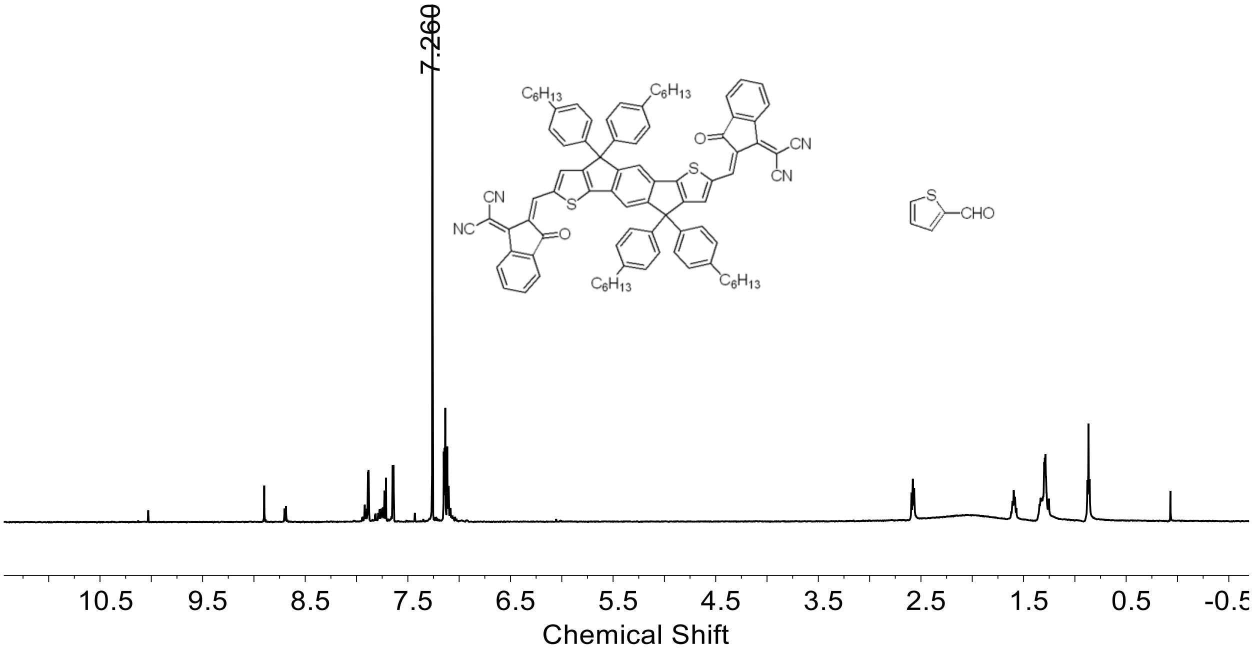

Figure S18. NMR of reaction between IDTIC and thiophene-2-carboxaldehye. It is a mixture of multiple reaction intermediates.

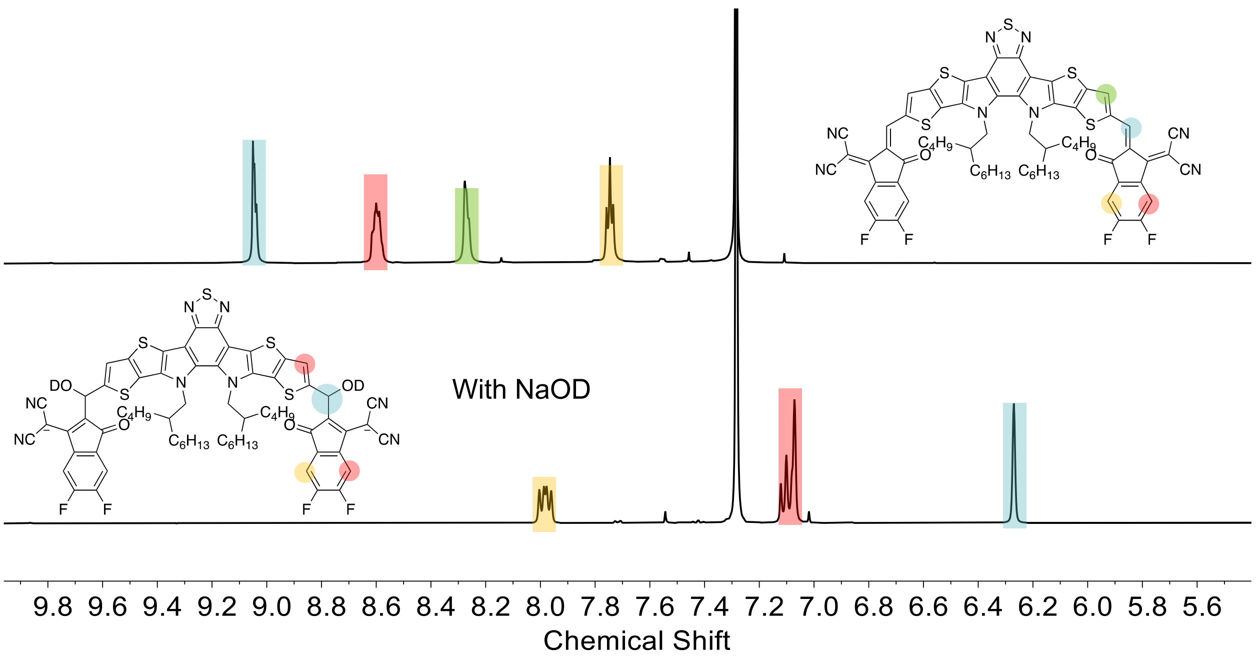

Figure S19. Stacked NMR spectra of original BTP-4F and its NaOD adduct in $CDCl_3$.

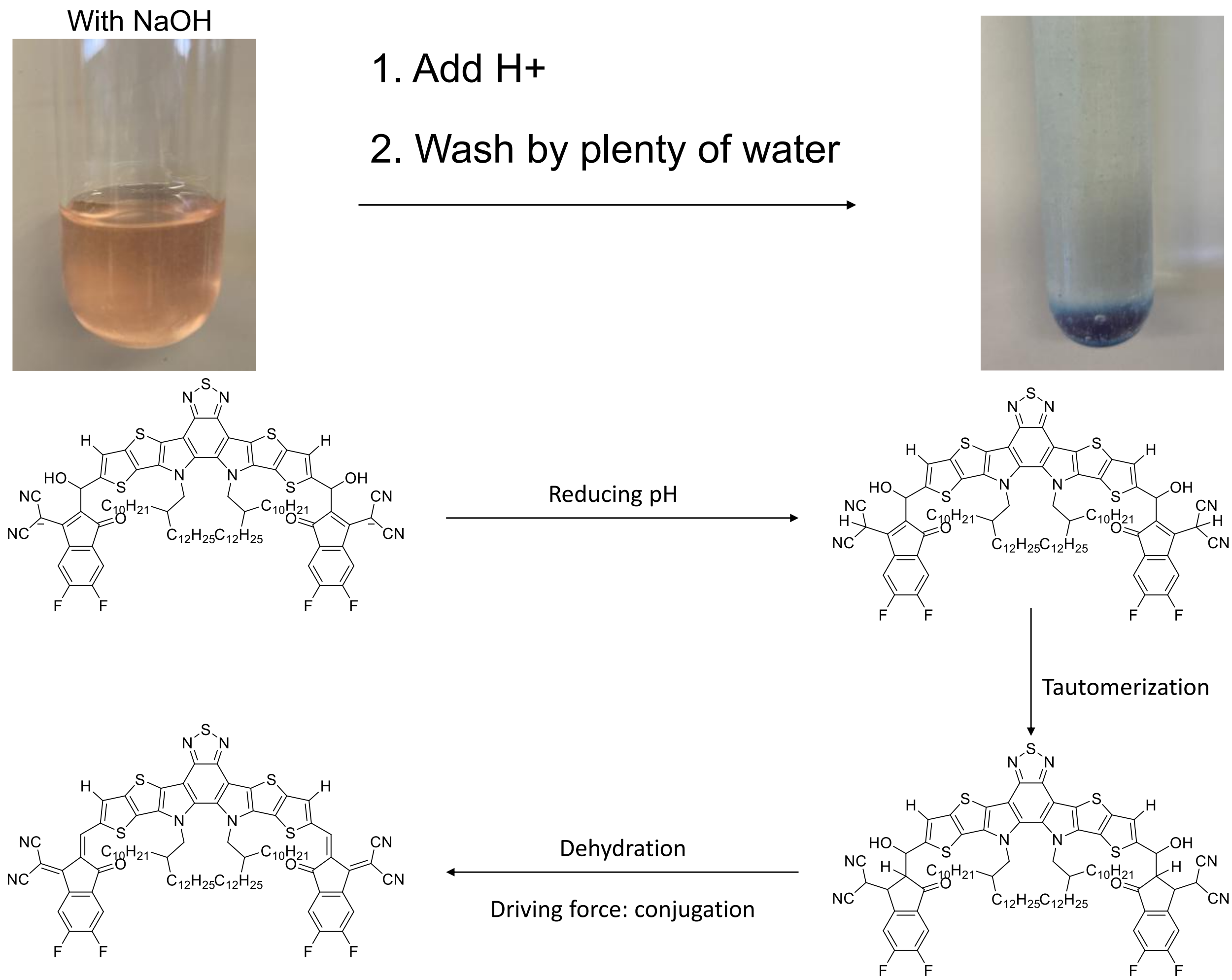

Figure S20. **Recovery of the NFA-NaOH adduct**. Reducing the pH through adding acid or more water will recover the NFA-NaOH, using DCM and water as solvent; proposed pathway of the recovery.

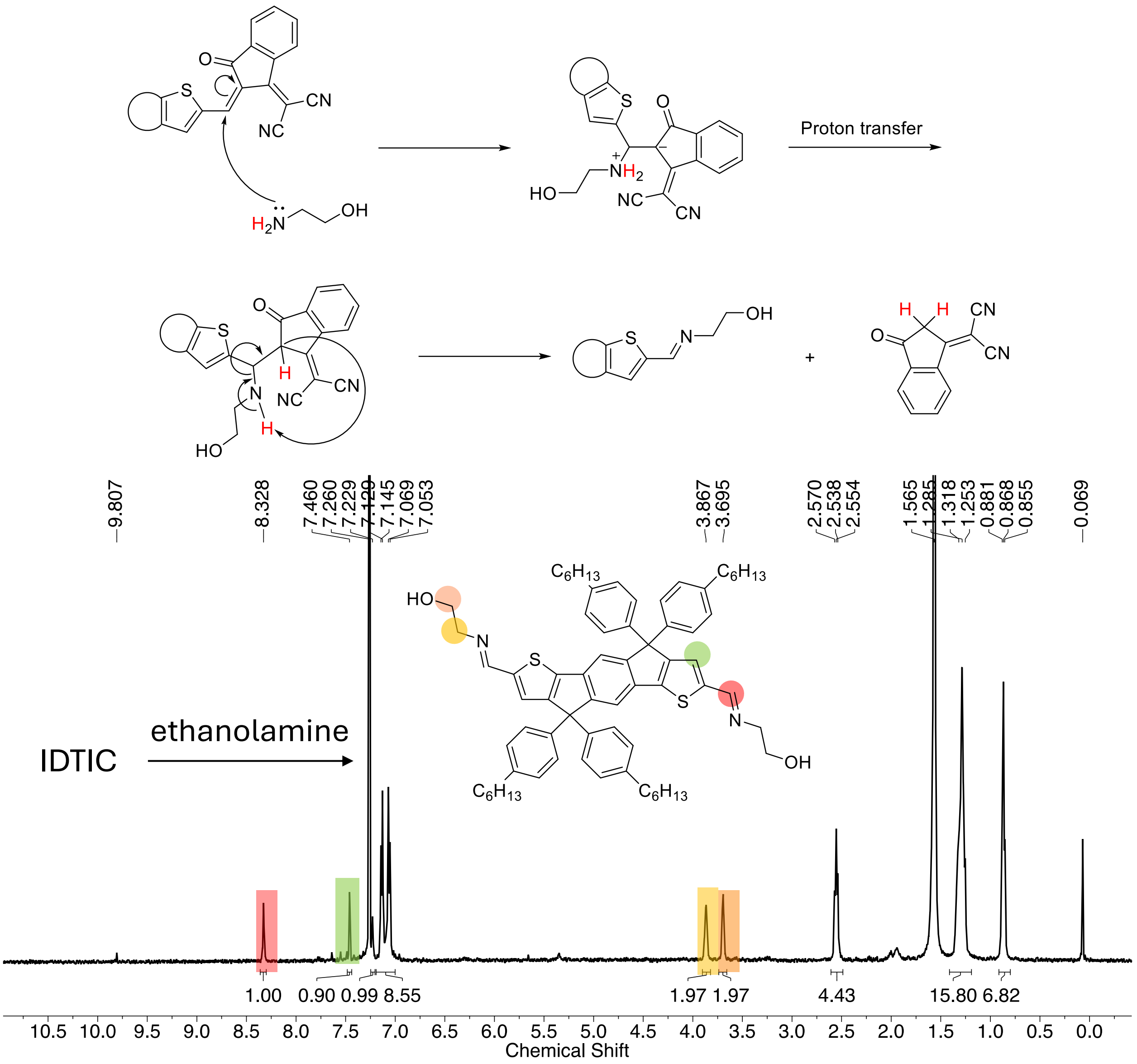

Figure S21. NMR of the product after treating IDTIC with excess ethanolamine, indicating the formation of imine. We were not able to observe the released IC unit in the NMR.

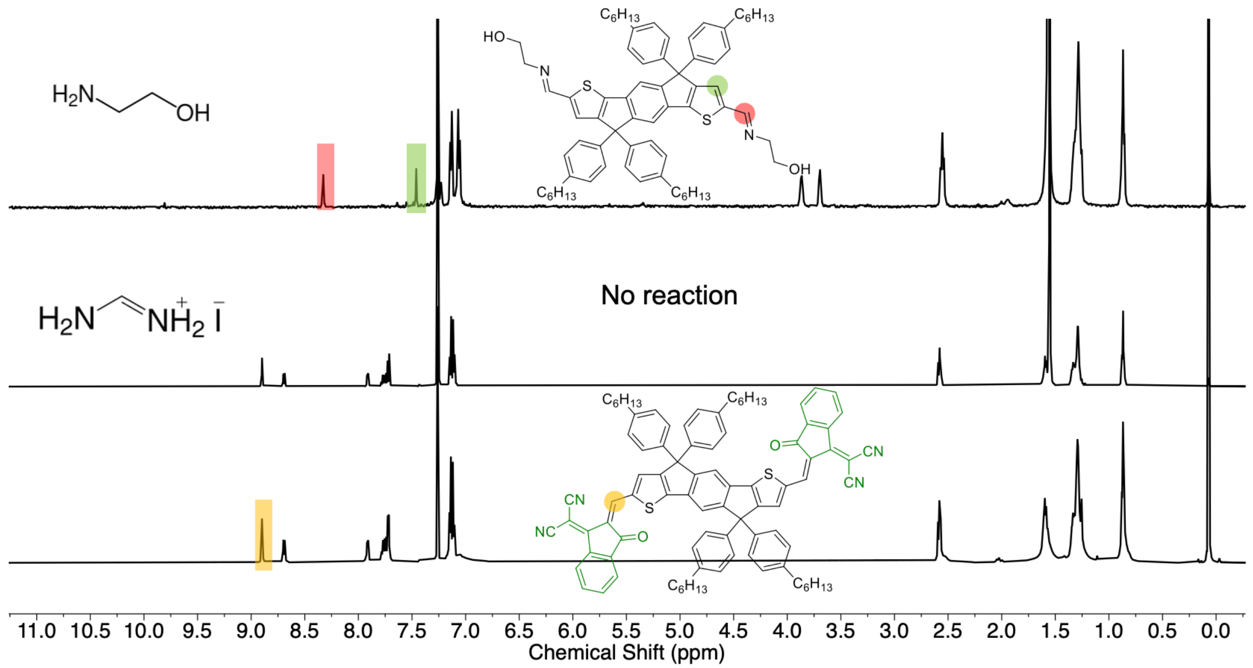

Figure S22. **Stacked NMR of the product after treating IDTIC with excess ethanolamine and FIA, indicating neutral or slightly acidic condition can suppress the reaction.**

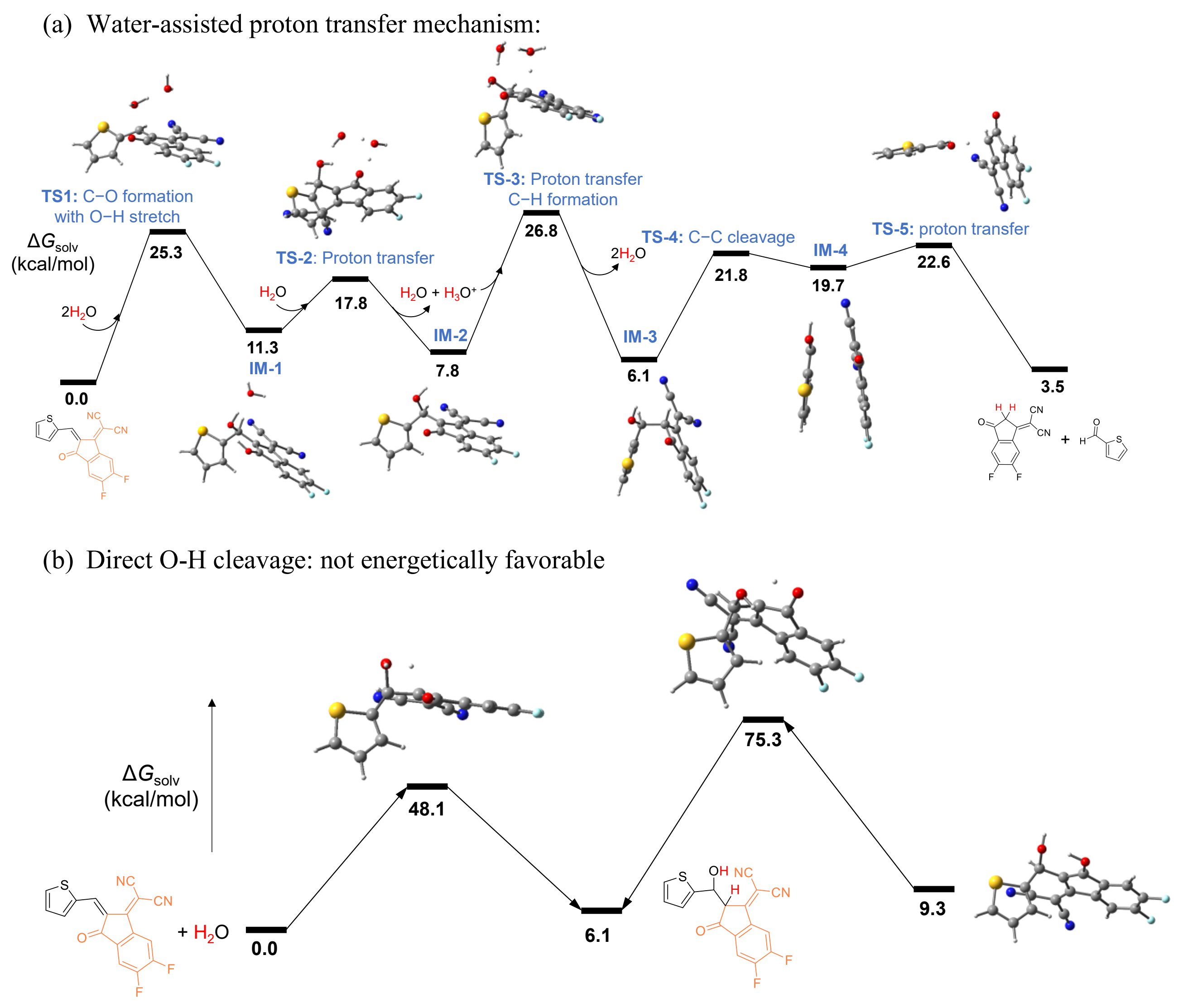

Figure S23. **Simulated energy diagram of decomposition of TFIC via direct O-H cleavage.** This process is not energetically favorable.

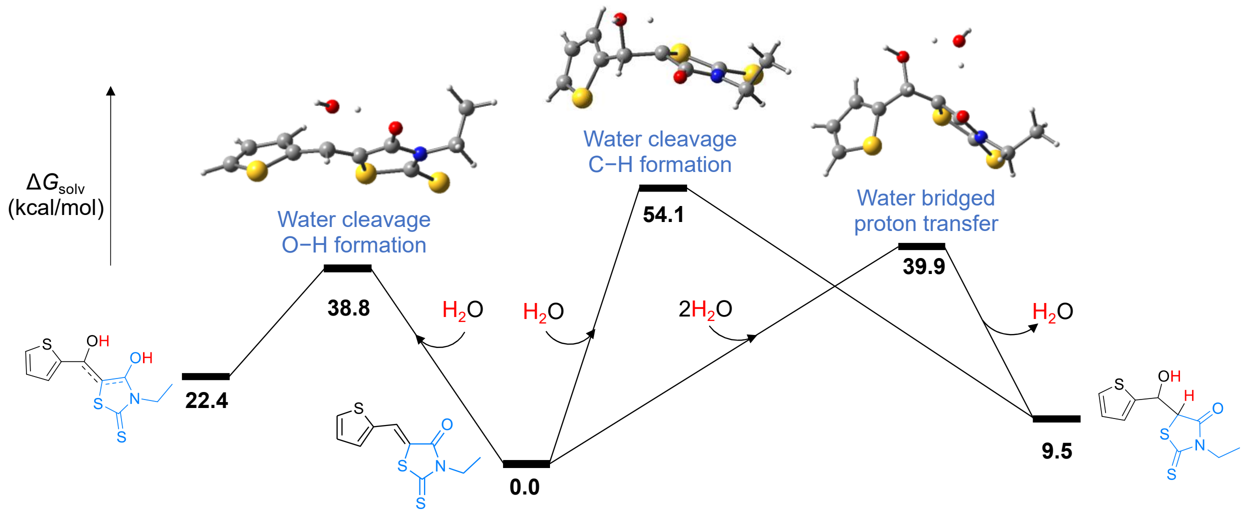

Figure S24. **simulated energy diagram of TRh decomposition**. This is not energetically favorable.

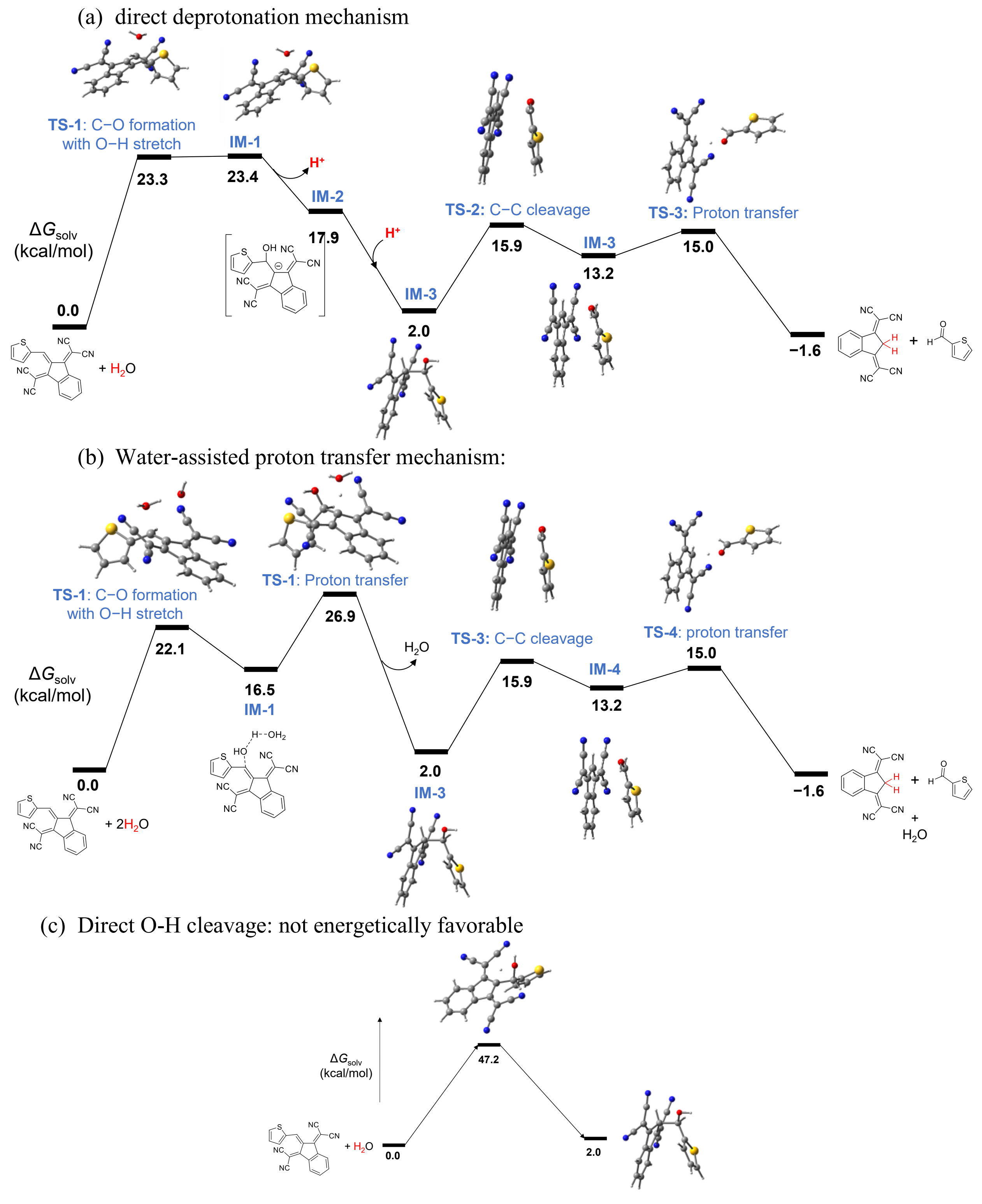

Figure S25. **Simulated energy diagram of T4CN.**

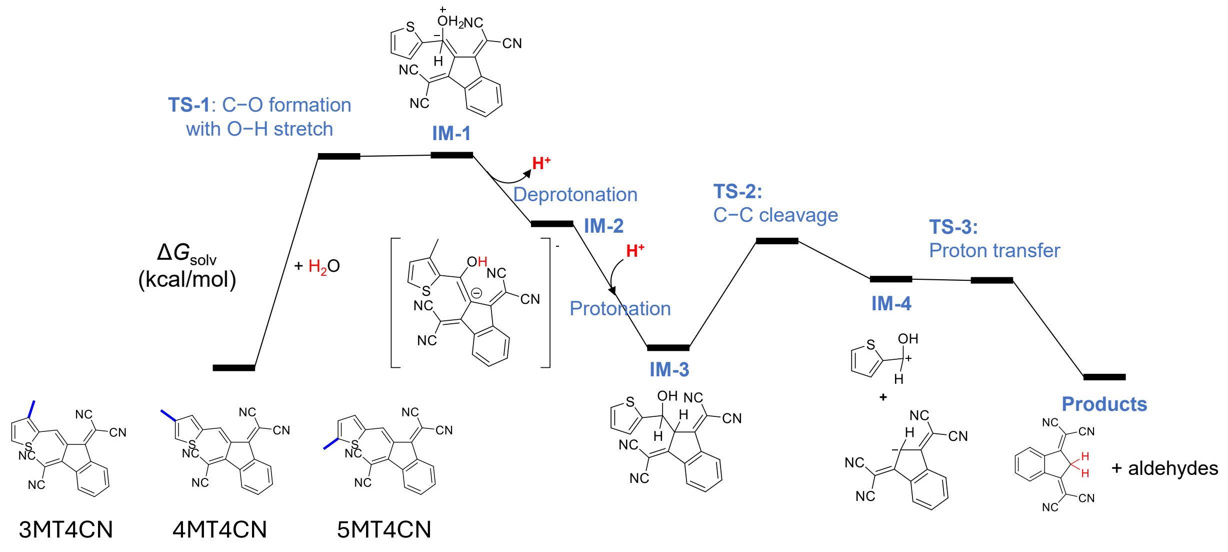

ΔG_{solv} (kcal/mol)	3MT4CN	4MT4CN	5MT4CN
TS-1	26.6	23.1	25.0
IM-1	27.2	23.3	25.2
IM-2	21.3	18.2	20.0
IM-3	5.5	2.1	4.4
TS-2	17.9	15.0	15.9
IM-4	13.4	11.9	11.8
TS-3	14.2	14.3	14.7
Products	-0.9	-1.4	-0.4

Figure S26. Simulated energy diagram of substituted T4CN, with representative energy diagram of 3MT4CN.

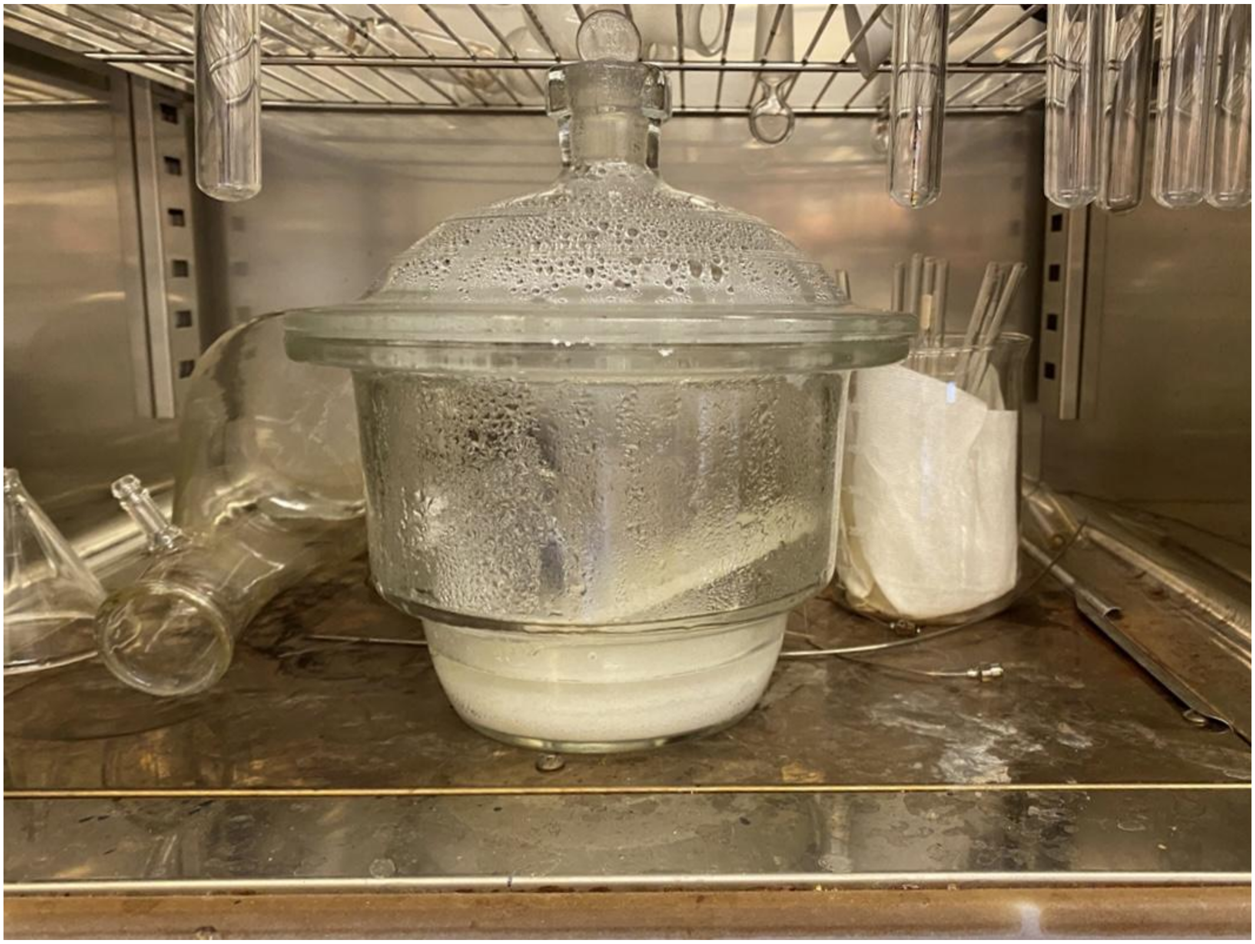

Figure S27. Home-made 85/85 environment, with saturated NaCl aq solution.

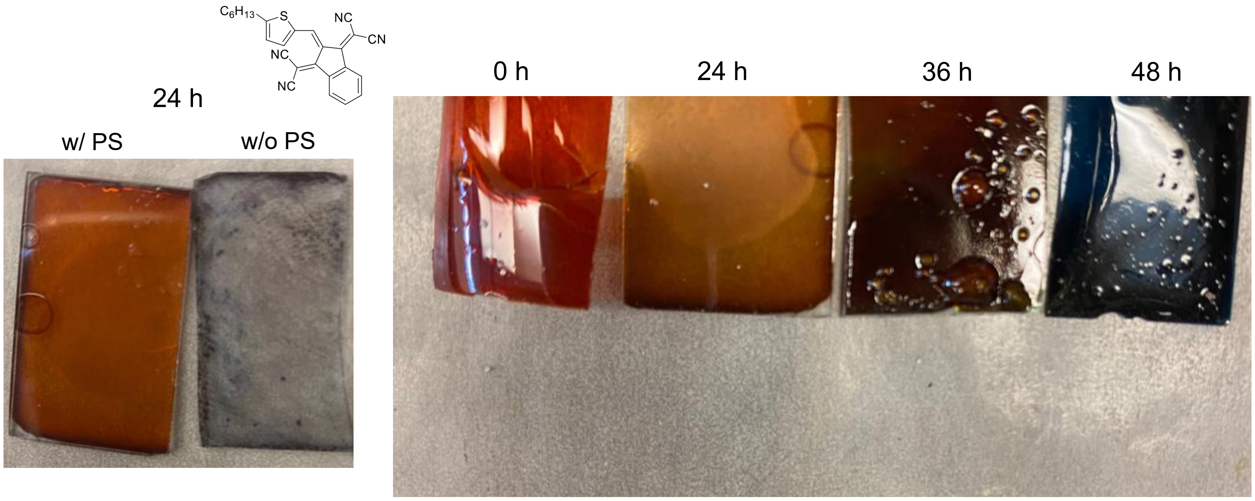

Figure S28. pictures of T4CN under 85/85 testing.

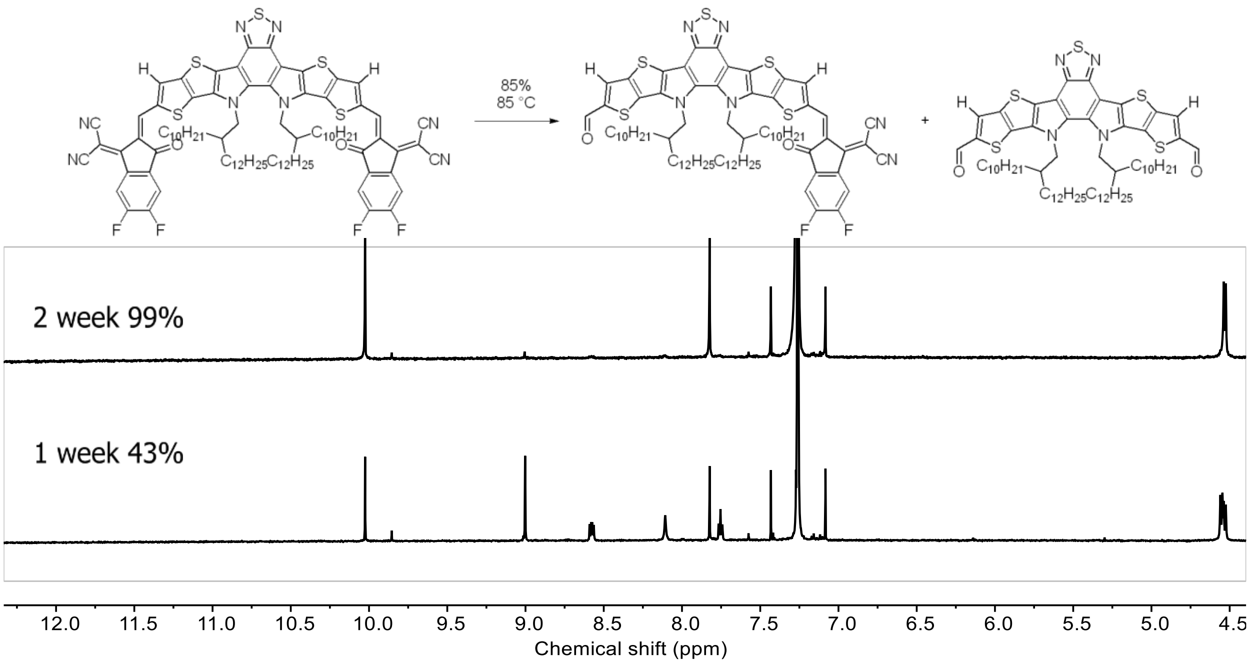

Figure S29. NMR of BTP4F under 85/85 testing condition.

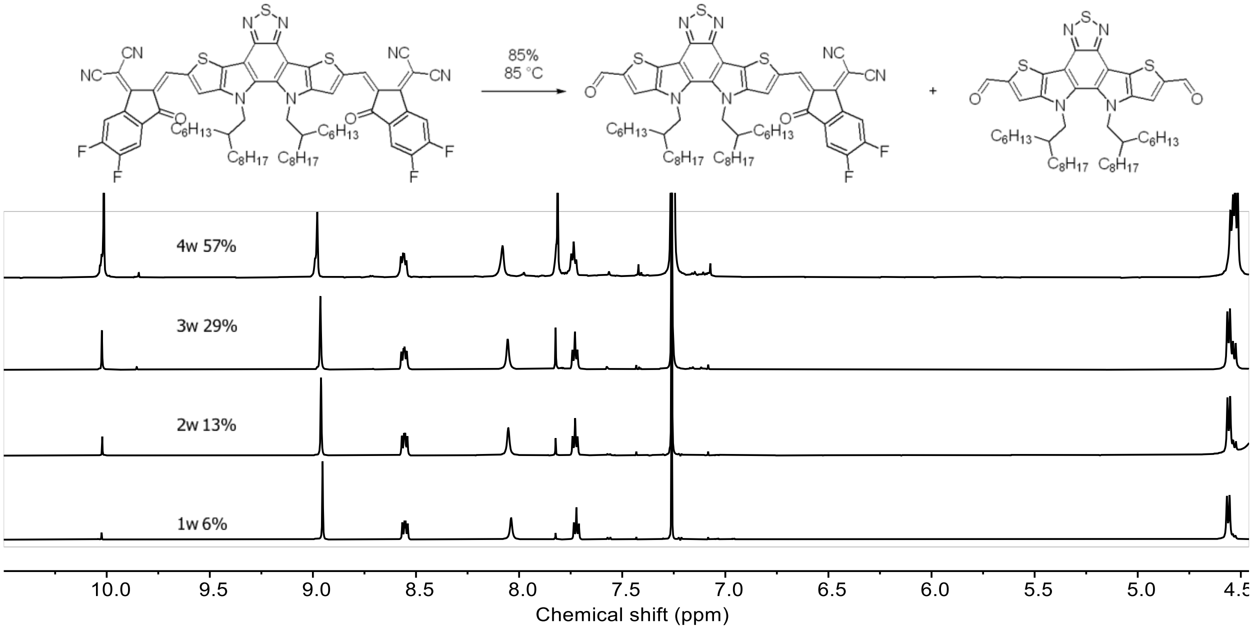

Figure S30. NMR of BZ4F under 85/85 testing condition.

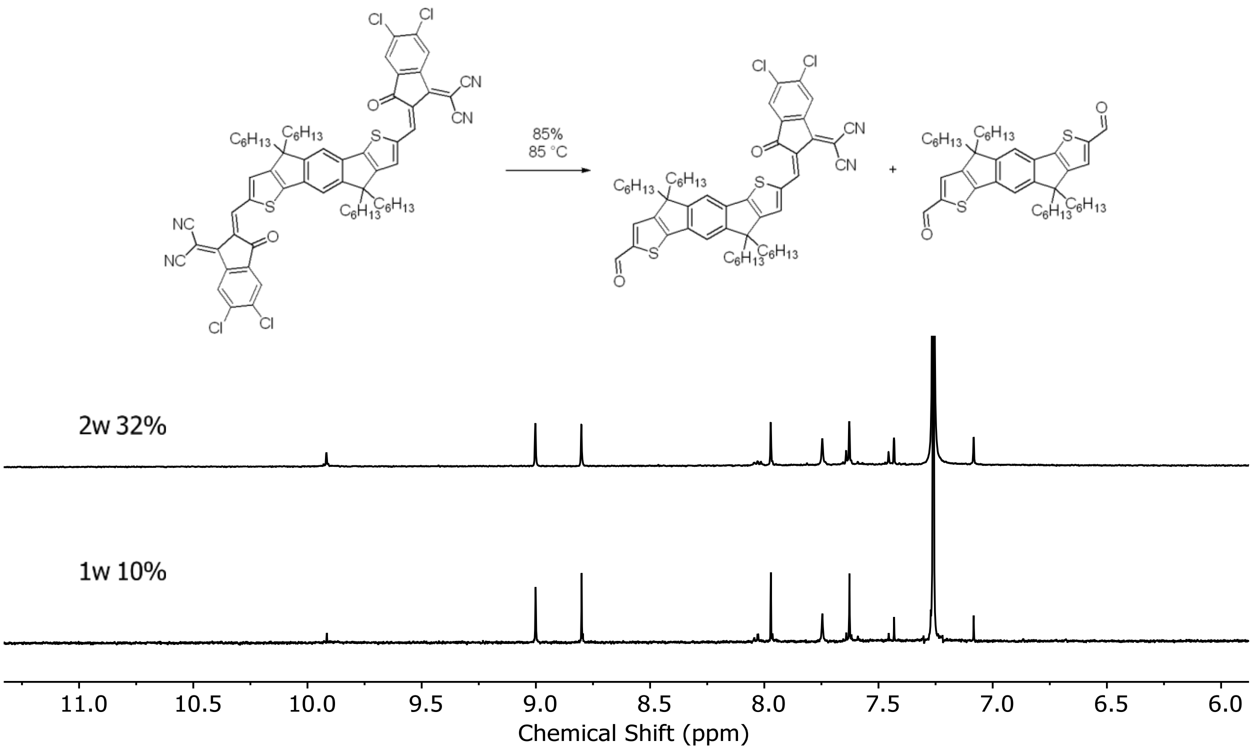

Figure S31. NMR of IDIC-4Cl under 85/85.

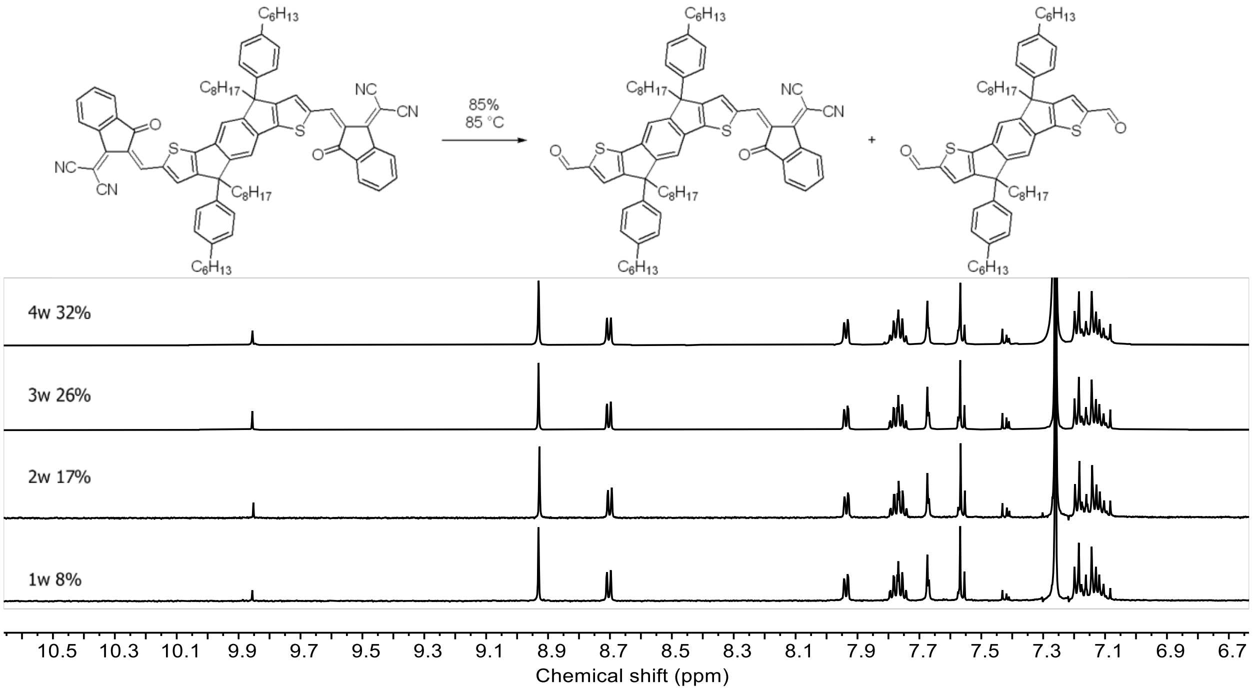

Figure S32. NMR of aIDIC under 85/85.

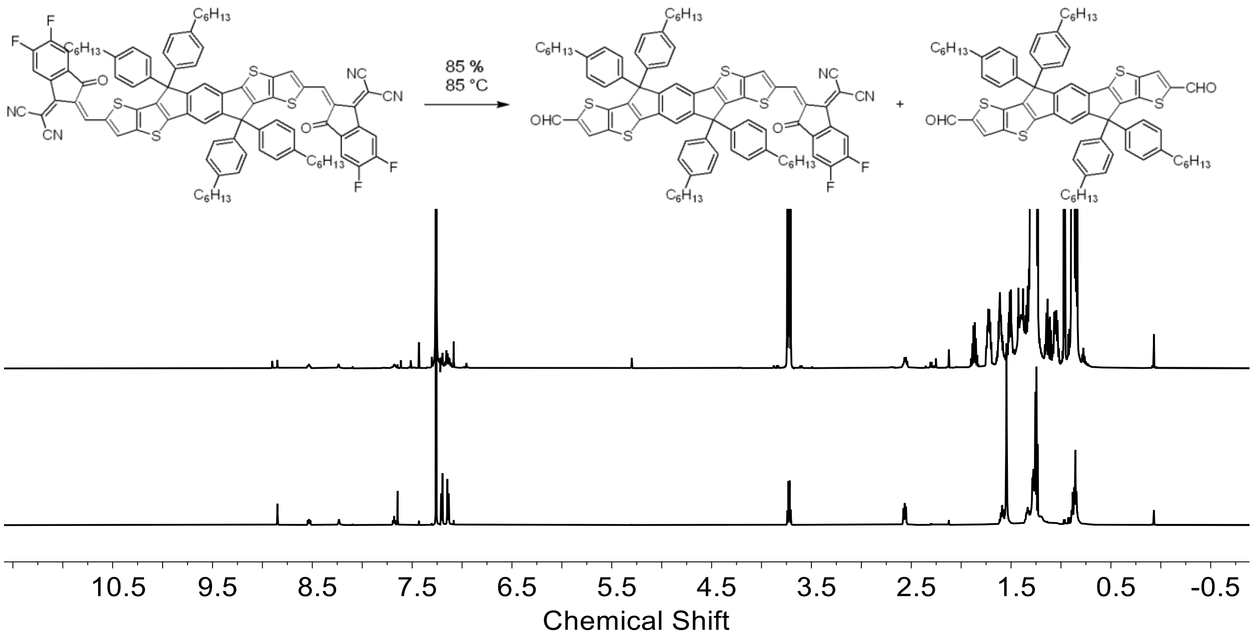

Figure S33. NMR of IT4F under 85/85 after one and two weeks.

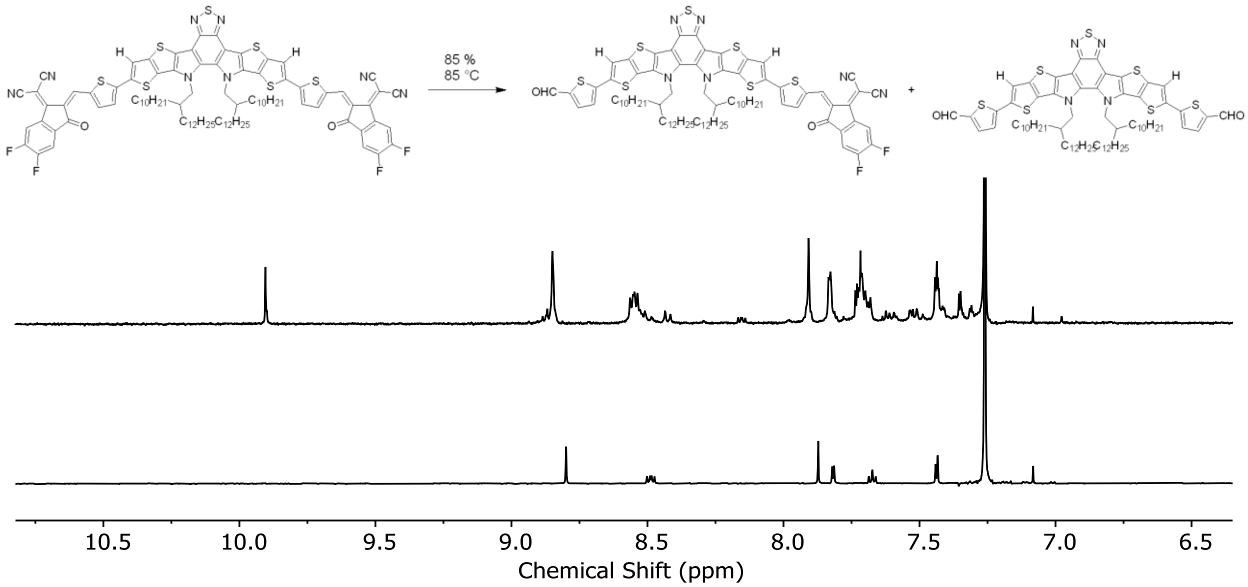

Figure S34. NMR of BTP-T-4F under 85/85 after 4 weeks.

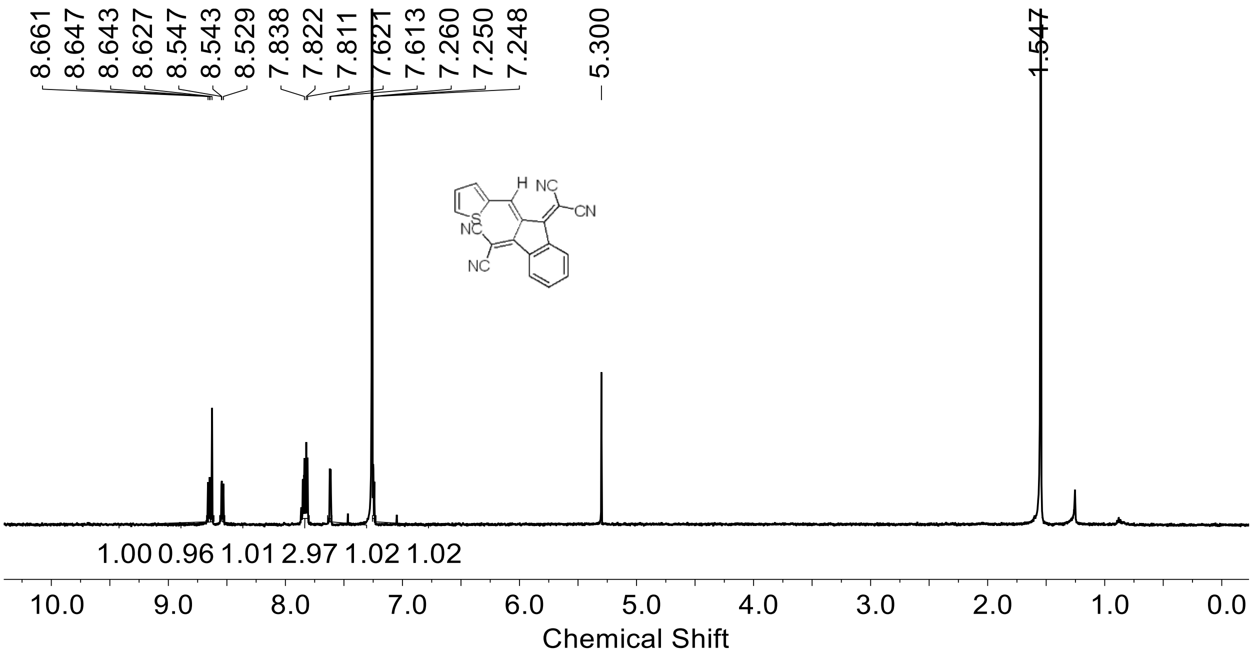

Figure S35. **NMR of T4CN in CDCl$_3$.**

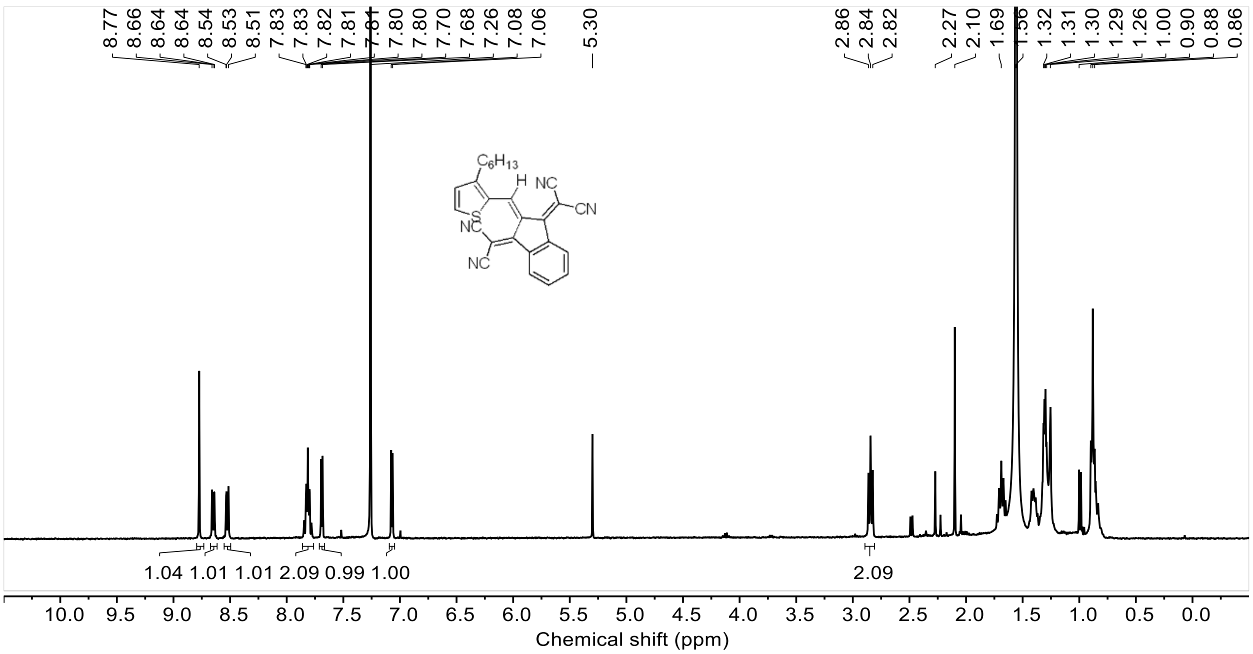

Figure S36. NMR of 3HT4CN in CDCl$_3$.

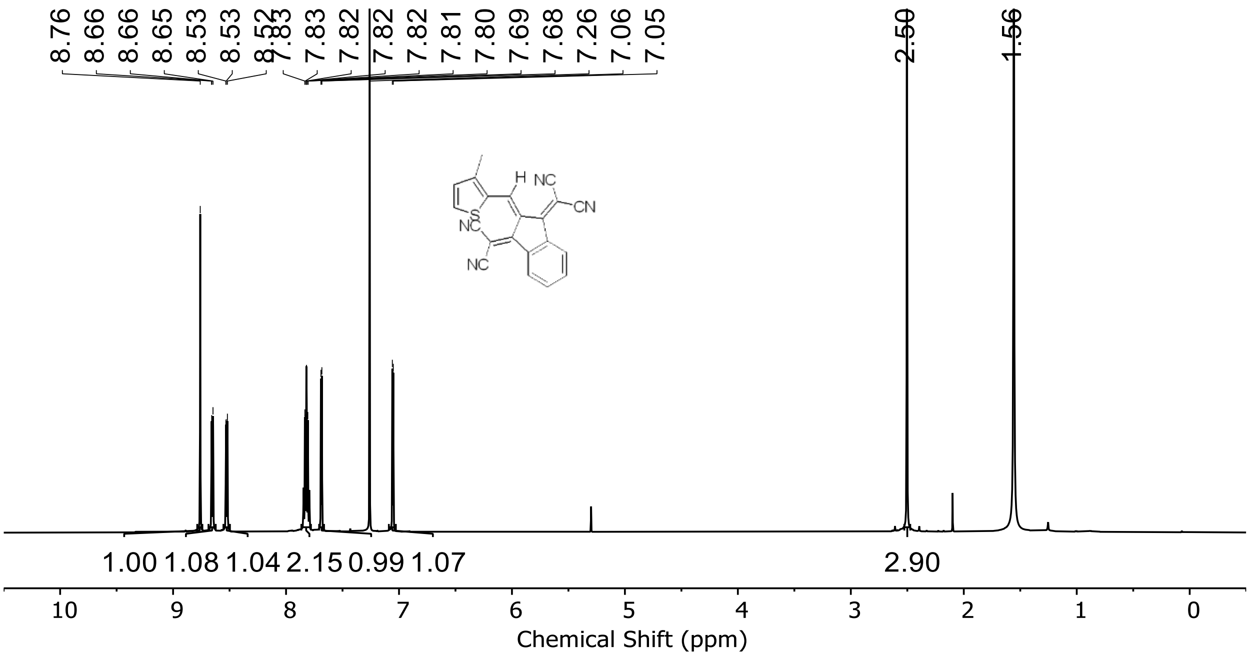

Figure S37. NMR of 3MT4CN in $CDCl_3$.

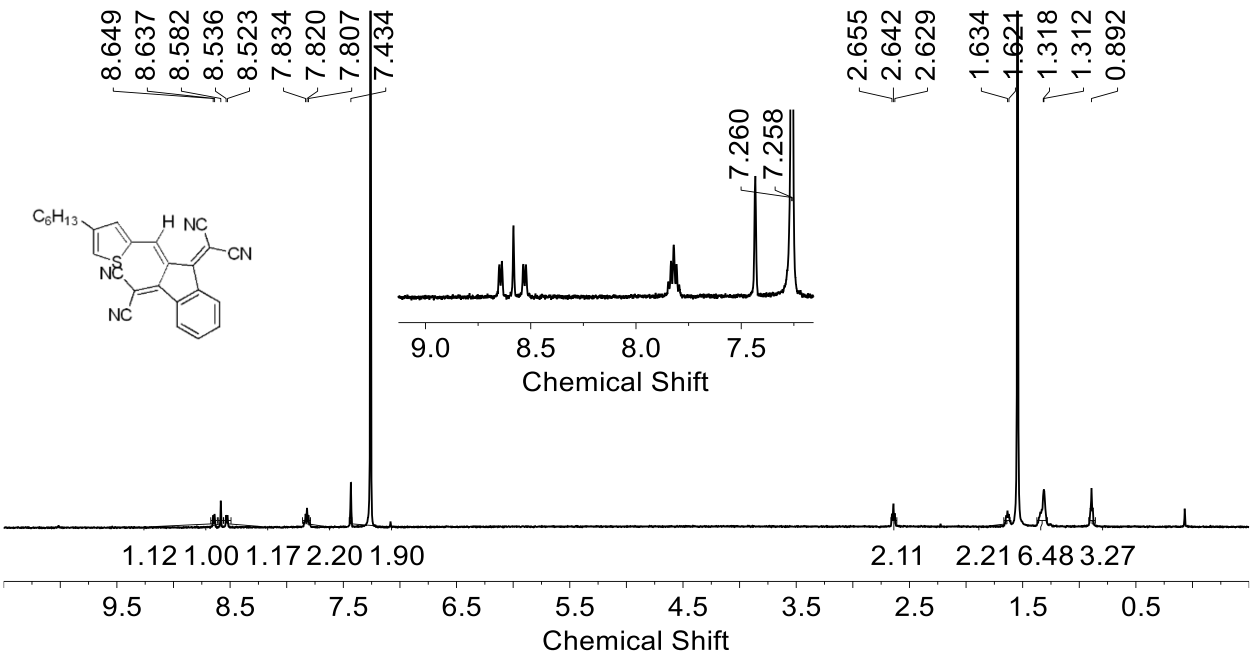

Figure S38. NMR of 4HT4CN in $CDCl_3$.

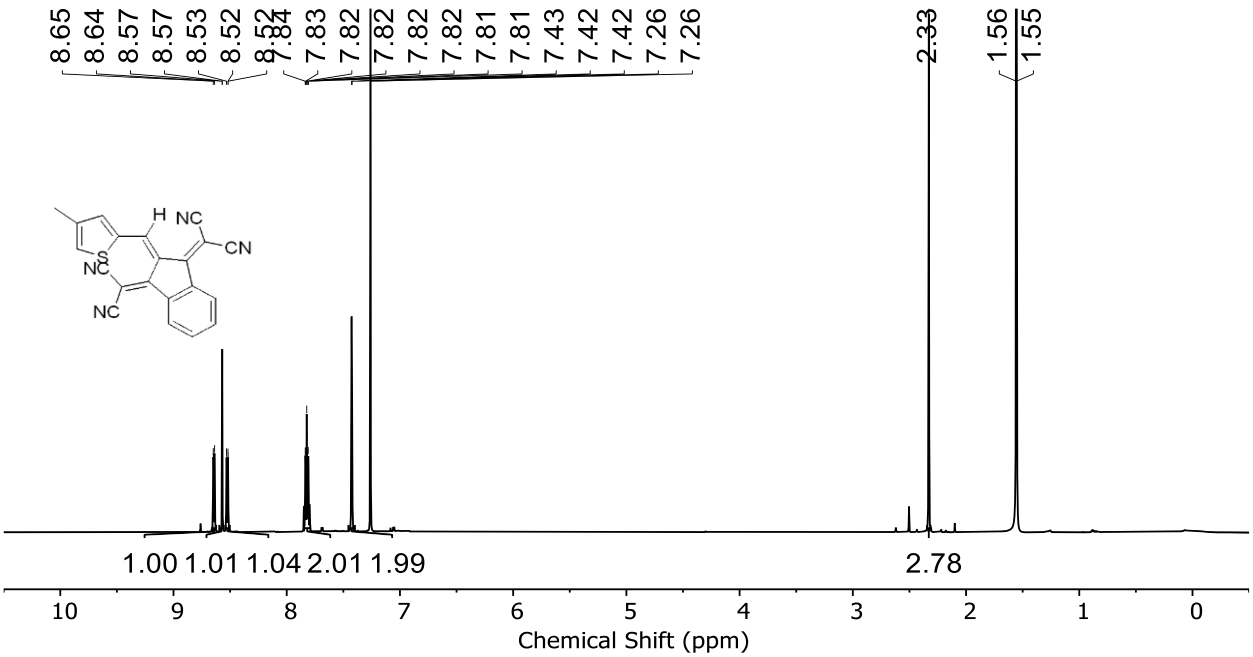

Figure S39. NMR of 4MT4CN in $CDCl_3$.

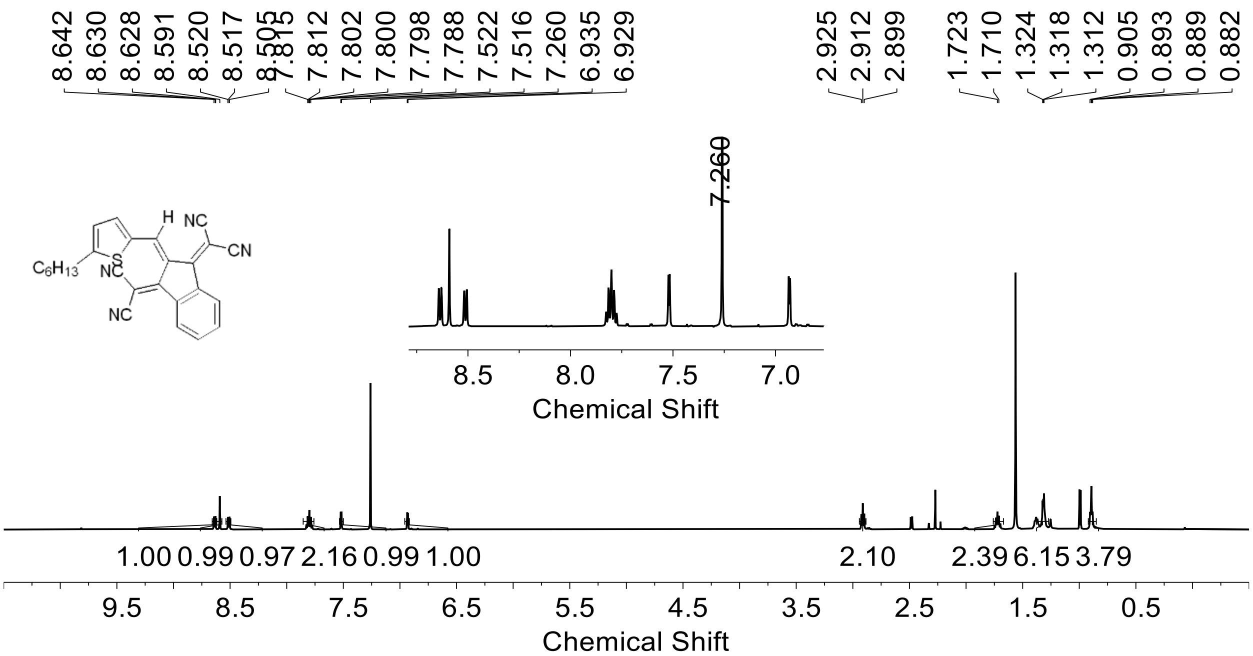

Figure S40. NMR of 5HT4CN in $CDCl_3$.

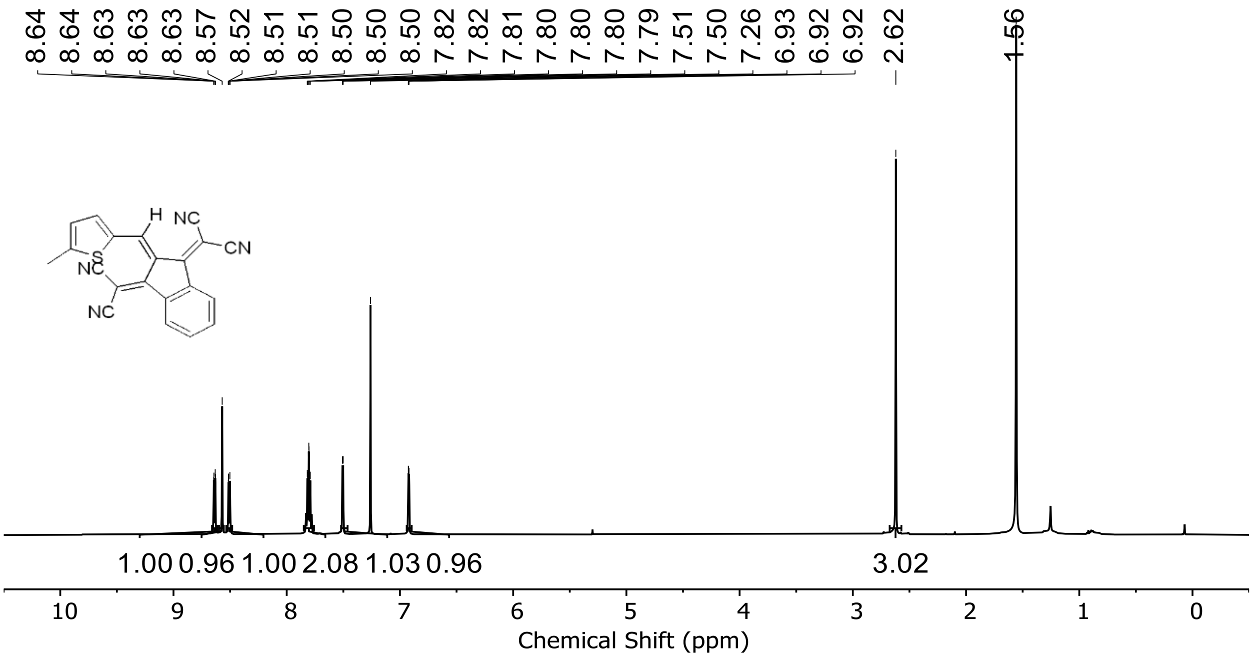

Figure S41. NMR of 5MT4CN in $CDCl_3$.

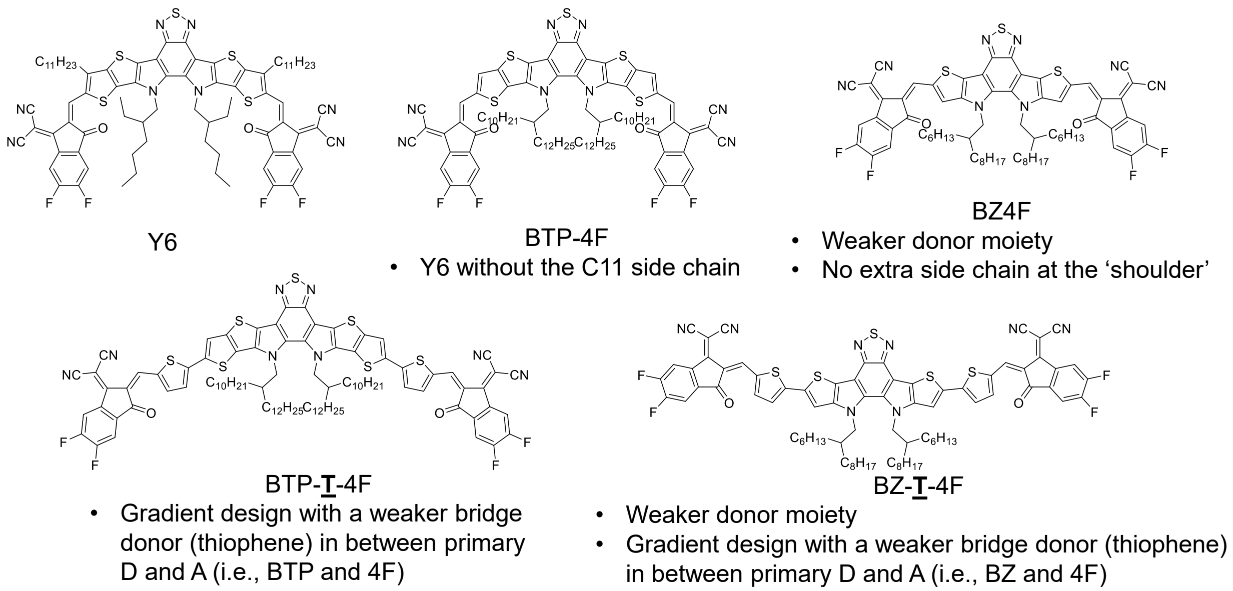

Figure S42. Structures of SMAs being tested in devices with their selection criteria.

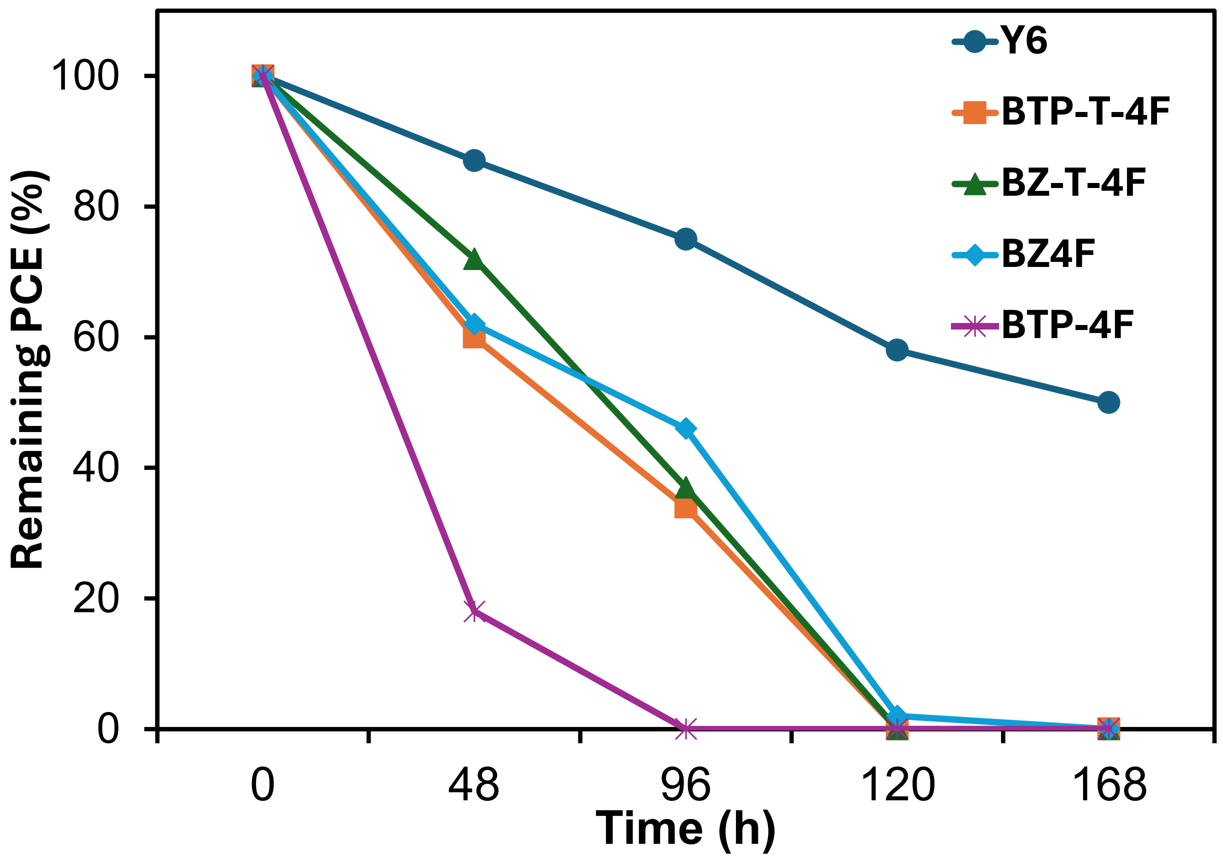

Figure S43. Device stability of five representative SMAs using D18-Cl as the donor polymer

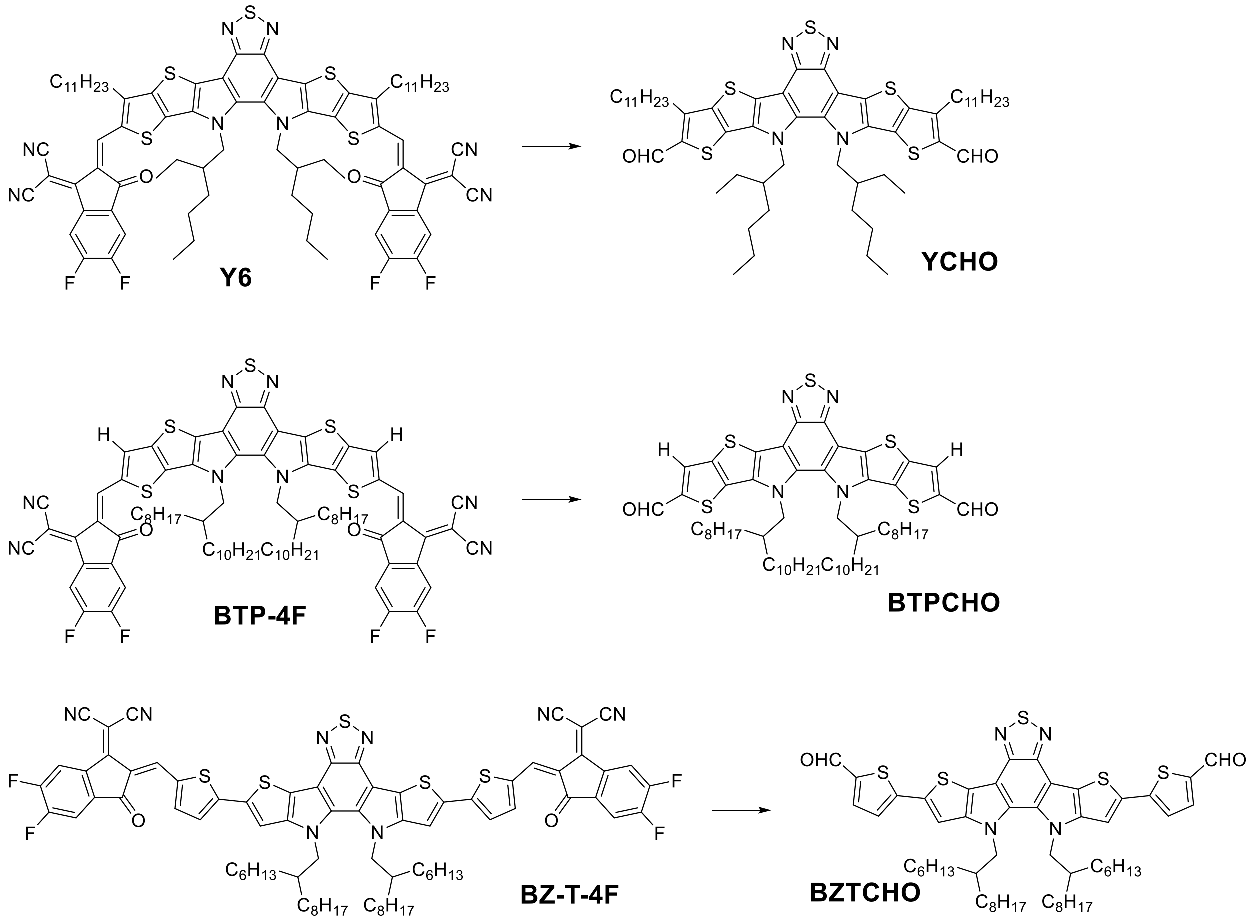

Figure S44. Structures of SMAs and their degraded products.

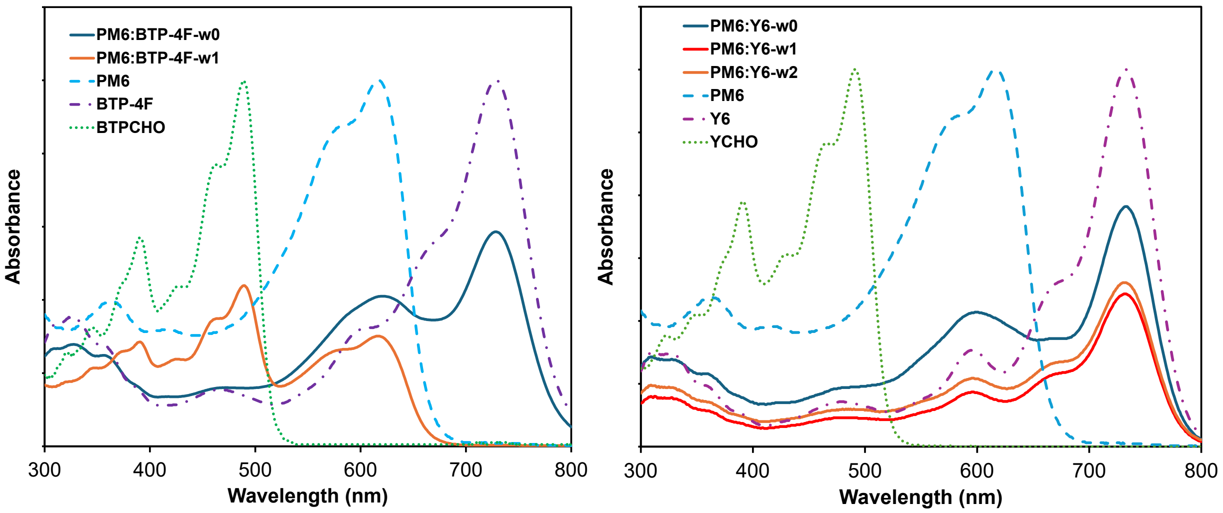

Figure S45. Solution-state UV-Vis absorption of active layers.

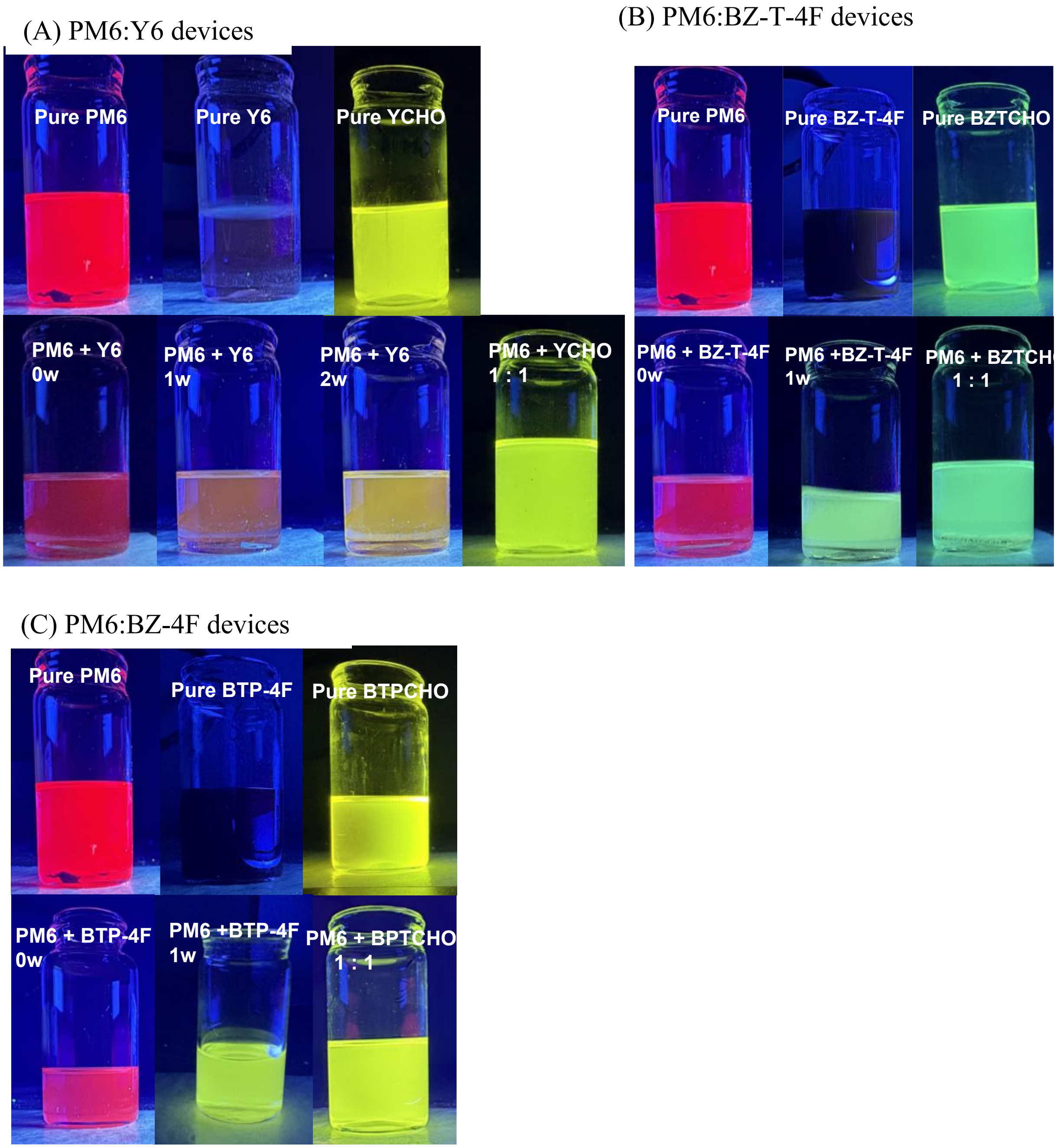

Figure S46. Fluorescence under 365 nm lamp of solution from re-dissolving active layers in chloroform. In all three cases, top row contains all pure compounds and the last image in the bottom row is the 'simulated' degradation mixture (assuming the specific SMA degraded into its corresponding aldehyde) where PM6 and the aldehyde were blended at 1:1 ratio. In all three cases, it was evident that the actual degraded mixture (after 2 weeks for PM6:Y6, and after one week for the other two cases) became more like the simulated degradation mixture (according to the fluorescence), indicating the formation of the aldehyde.

Table S1. Device performances

System	Time	V_{OC} (V)	J_{SC} (mA · cm^2)	FF (%)	PCE (%)
**PM6:Y6**	0d	0.731 (0.727 ± 0.006)	25.98 (25.52 ± 0.40)	67.98 (67.00 ± 0.55)	12.91 (12.43 ± 0.27)
	7d	0.618 (0.613 ± 0.007	19.68 (19.24 ± 0.47)	46.46 (45.84 ± 0.65)	5.65 (5.41 ± 0.261)
**PM6:BTP-4F**	0d	0.806 (0.802 ± 0.002)	14.38 (14.05 ± 0.25)	59.67 (59.36 ± 0.29)	6.91 (6.69 ± 0.14)
	7d	-	-	-	-
**PM6:BZ-T-4F**	0d	0.814 (0.802 ± 0.008)	15.59 (15.40 ± 0.36)	58.34 (58.06 ± 0.27)	7.40 (7.17 ± 0.24)
	7d	-	-	-	-
**D18Cl:Y6**	0h	0.823 (0.820 ± 0.005)	25.76 (25.39 ± 0.45)	64.39 (63.86 ± 0.58)	13.65 (13.30 ± 0.33)
	48h	0.791 (0.782 ± 0.008)	24.10 (23.95 ± 0.15)	62.28 (61.93 ± 0.29)	11.87 (11.60 ± 0.22)
	96h	0.786 (0.779 ± 0.006)	20.89 (19.92 ± 0.63)	62.46 (61.89 ± 0.37)	10.26 (9.61 ± 0.41)
	120h	0.729 (0.719 ± 0.009)	19.03 (18.65 ± 0.38)	58.09 (57.69 ± 0.32)	8.05 (7.73 ± 0.22)
	168h	0.735 (0.719 ± 0.013)	16.88 (16.11 ± 0.69)	56.56 (55.91 ± 0.53)	7.02 (6.48 ± 0.44)
**D18Cl:BTP-4F**	0h	0.600 (0.576 ± 0.023)	3.64 (3.49 ± 0.38)	34.44 (33.86 ± 0.41)	0.75 (0.68 ± 0.08)
	48h	0.230 (0.209 ± 0.021)	2.10 (1.74 ± 0.29)	29.41 (29.10 ± 0.24)	0.14 (0.11 ± 0.03)
	96h	-	-	-	-
	120h	-	-	-	-
	168h	-	-	-	-
**D18Cl:BTP-T-4F**	0h	0.753 (0.706 ± 0.037)	8.65 (8.35 ± 0.36)	39.09 (38.51 ± 0.46)	2.55 (2.27 ± 0.16)
	48h	0.729 (0.712 ± 0.019)	6.63 (6.22 ± 0.39)	37.65 (37.03 ± 0.47)	1.82 (1.64 ± 0.14)
	96h	0.618 (0.596 ± 0.018)	4.55 (4.01 ± 0.40)	33.53 (33.18 ± 0.39)	0.94 (0.80 ± 0.11)
	120h	-	-	-	-
	168h	-	-	-	-
**D18Cl:BZ4F**	0h	0.850 (0.824 ± 0.043)	5.30 (4.74 ± 0.42)	35.46 (35.05 ± 0.47)	1.60 (1.37 ± 0.14)
	48h	0.780 (0.774 ± 0.009)	4.06 (3.85 ± 0.17)	31.51 (31.15 ± 0.26)	1.00 (0.93 ± 0.05)
	96h	0.663 (0.655 ± 0.014)	3.68 (3.48 ± 0.20)	30.39 (29.37 ± 0.86)	0.74 (0.67 ± 0.05)
	120h	0.180 (0.171 ± 0.012)	0.63 (0.58 ± 0.05)	26.76 (26.20 ± 0.80)	0.03 (0.03 ± 0.01)
	160h	0.206 (0.139 ± 0.077)	0.57 (0.56 ± 0.06)	26.14 (26.05 ± 0.17)	0.03 (0.02 ± 0.01)
**D18Cl:BZ-T-4F**	0h	0.816 (0.807 ± 0.009)	16.76 (16.38 ± 0.43)	55.16 (54.83 ± 0.40)	7.55 (7.25 ± 0.22)
	48h	0.694 (0.691 ± 0.003)	16.82 (16.58 ± 0.23)	39.23 (38.71 ± 0.67)	4.58 (4.43 ± 0.13)
	96h	0.440 (0.426 ± 0.011)	15.83 (15.42 ± 0.29)	36.92 (36.31 ± 0.45)	2.57 (2.39 ± 0.12)
	120h	-	-	-	-
	160h	-	-	-	-
